# Late‐Stage Functionalization of Peptides on the Solid Phase

**DOI:** 10.1002/anie.4556652

**Published:** 2026-06-10

**Authors:** Marius Werner, Truc Lam Pham, Franziska Thomas

**Affiliations:** ^1^ Institute of Organic Chemistry Heidelberg University Heidelberg Germany; ^2^ Institute of Pharmacy and Molecular Biotechnology (IPMB) Heidelberg University Heidelberg Germany; ^3^ Research School of Chemistry Australian National University Acton Australia

**Keywords:** late‐stage functionalization, non‐canonical amino acids, peptide therapeutics, peptides, solid phase peptide synthesis

## Abstract

Chemical modification is a means of tailoring the structure and function of peptides. This is of particular interest in the field of peptide drug development. Methods that enable straightforward modification of peptides with non‐canonical residues are therefore in high demand. On‐resin late‐stage functionalization is a powerful, cost‐ and time‐efficient tool for modifying peptides. It also allows for high‐throughput experimentation, making it well suited to streamlining the development of peptide therapeutics. In this minireview, we provide an overview of the chemical approaches for on‐resin late‐stage peptide functionalization , most of which have been developed over the past decade. These reactions range from nucleophilic substitution and carbonyl chemistry to metal catalysis and photocatalysis. Many of the presented approaches have been shown to be compatible with short and long peptides, and to modulate their bioactivity. Impressive examples of peptide library synthesis in high‐throughput formats demonstrate the enormous potential of late‐stage peptide functionalization for the development of peptide therapeutics in the years to come.

## Graphical abstract

Peptide modifications are essential to control pharmacodynamic and pharmacokinetic properties of peptide drugs. Consequently, strategies that allow for efficient and rapid incorporation of non‐canonical modifications into peptides in parallel formats are highly sought after. This minireview describes the recent advances of on‐resin late‐stage functionalization of peptides and provides an overview of the scope and potential of these approaches.

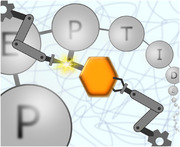

## 1 Introduction

Peptides have long been considered an important class of therapeutics due to their wide range of functions [1, 2, 3, 4]. Their medium size, in particular, fills the gap between small molecules and biologics [5]. Compared to small molecules, they show an improved specificity, and they have better cell permeability and lower immunogenicity than biologics. Another advantage is that they are easily accessible via highly modular solid‐phase peptide synthesis (SPPS). The first and best‐known example of a peptide drug is insulin, which was approved in the 1920s to treat type 1 diabetes [6]. Since then, however, only a relatively small number of new peptide therapeutics have been approved. The main challenge in developing new peptide drugs is the low biostability of natural peptides, but this obstacle can be overcome through chemical modification [7, 8].

**FIGURE 1:**
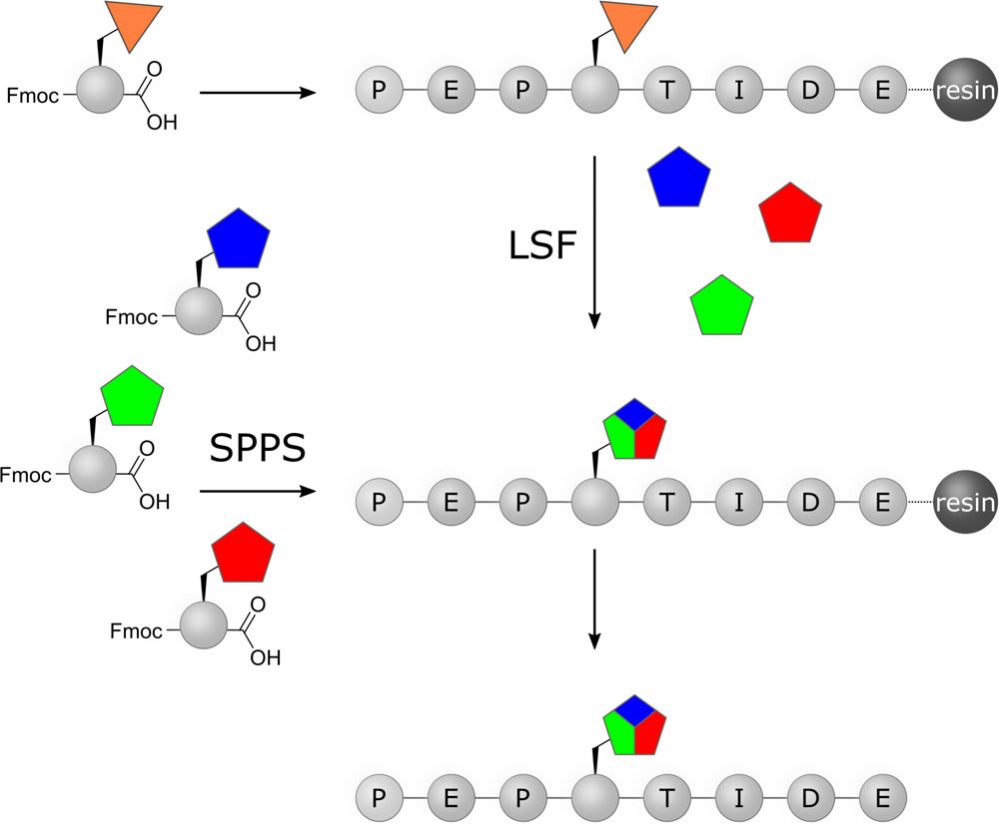
Concept of on‐resin LSF compared to traditional SPPS with non‐canonical amino acid building blocks.

The traditional method of chemically modifying peptides involves synthesizing SPPS‐compatible building blocks from non‐natural amino acids [7]. Although this strategy permits the use of a wide range of chemical reactions to produce the building blocks, it is also highly labor‐intensive. Furthermore, only a limited number of non‐canonical amino acid building blocks are commercially available, and they are expensive. To create libraries of peptides with non‐canonical modifications in a cost‐ and time‐efficient manner, alternative approaches are required. One such strategy is the site‐selective modification of peptides after the peptide chain has been assembled. Ideally, this would be carried out in an aqueous solution on unprotected peptides using biorthogonal chemistry. However, chemical reactions that meet biorthogonality criteria are limited. A compromise that allows for a wide range of chemical reactions and straightforward, efficient peptide diversification is the late‐stage functionalization (LSF) of resin‐bound peptides (Figure 1). In this approach, an amino acid containing a reactive handle is introduced into the peptide sequence via SPPS. This can be either a canonical amino acid or an inexpensive, ideally commercially available, non‐canonical amino acid building block. This residue is then selectively modified by a variety of substrates, serving as a starting point for further diversification. Since LSF is performed on resin‐bound peptides that remain protected, a wide range of chemical reactions are tolerated, including those that occur under harsher conditions and in non‐aqueous solvents. Site‐selectivity is achieved through orthogonal protecting group strategies [9]. Thus, on‐resin LSF enables rapid and extensive access to chemical and structural diversity from a single starting material.

Pioneering work on the LSF of resin‐bound peptides was described 20–30 years ago [10, 11, 12, 13, 14, 15, 16, 17, 18, 19, 20, 21, 22]. These early examples primarily focused on introducing natural post‐translational modifications, such as glycosylation and phosphorylation [10, 11, 12, 13]. Although examples of the introduction of non‐natural modifications were reported back then, there has been rising interest over the past decade in developing methods to introduce non‐canonical modifications into peptides at a late stage of synthesis, in recognition of their great potential for developing new peptide therapeutics [1, 2, 3, 4, 5, 7, 8]. As this is a relatively new trend, many reports focus on method development, and few have described its application in developing peptide therapeutics in parallel formats. However, all the methods described have the potential to be used in peptide diversification and thus in the development of peptide therapeutics. This minireview aims to highlight recent advances in this field and provide an overview of the chemical reactions that have been established for on‐resin LSF. We focus primarily on side chain modifications. Backbone modifications have mainly been performed in solution and are covered in detail elsewhere [23, 24]. Only a few on‐resin modifications of the peptide backbone have been described, and these are reviewed herein. Peptide macrocyclization and stapling have attracted considerable attention in the past, and numerous reviews on these topics have been published [25, 26, 27, 28, 29, 30, 31, 32, 33, 34, 35]. Therefore, we will only include examples of on‐resin peptide macrocyclization or stapling if they involve structural diversification beyond cyclization. We have structured the review according to the reaction classes, starting with nucleophilic substitution, followed by carbonyl chemistry and metal‐catalyzed reactions, redox reactions, pericyclic reactions, and photocatalysis. We have also included a short opening chapter to this review in which we outline some general considerations regarding on‐resin LSF. Before concluding the review, we provide a brief assessment of the potential of resin‐based LSF for the development of peptide therapeutics. In the Supporting Information, we provide some practical information on performing LSF on resin‐bound peptides.

## 2 General Considerations in On‐Resin Late‐Stage Functionalization of Peptides

The heterogeneity of LSF on the solid phase makes identifying the optimal synthesis conditions challenging. Conditions optimized for chemical reactions in solution cannot be applied directly to the solid phase, and numerous parameters must be considered. Below, we briefly discuss aspects of resin selection and protecting group strategies. More detailed information can be found in the Supporting Information.

### 2.1 Resins and Linkers

In solid‐phase synthesis, the swollen resin is considered the solvent. Consequently, the swelling behavior and polarity of a resin greatly influence chemical reactions carried out on resin, and must therefore be considered when developing new LSF methods. Polystyrene (PS) resin is most commonly used due to its cost‐effectiveness. However, it is highly nonpolar, resulting in good swelling in nonpolar solvents but poor swelling in alcohols and water. Conversely, polyethylene glycol (PEG) and polyacryl‐amide resins exhibit greater polarity and improved swelling in many solvents than PS resin, but have a lower loading density. While this is advantageous for synthesizing longer peptides, it can cause problems during scaling up. In general, solvents that induce strong swelling of the resin are better suited to synthesis. However, this is not a mandatory requirement as long as swelling is observed. For example, the amination of peptides containing iodohomoalanine on PS resin is more effective in acetonitrile than in dimethylformamide (DMF), although resin swelling is lower in acetonitrile [36]. Switching from PS resin to the higher‐swelling PEG resin does not necessarily lead to a higher yield. PS resin has been shown to give better yields in photocatalytic decarboxylative arylation than PEG resin [37], yet both types of resin can be used for the hydroboration of peptides modified with alkenes and alkynes [38].

The reaction conditions used in an LSF are determined by the peptide linkage to the resin and the protecting group strategy employed in SPPS [39, 40, 41]. The 9‐fluorenylmethoxycarbonyl (Fmoc)/*tert*‐butyl (*t*‐Bu) strategy is typically used, relying on acid‐sensitive side chain protecting groups and acid‐sensitive linkers. Therefore, basic reaction conditions are generally unproblematic, whereas acidic conditions cannot be considered. The Wang and Rink amide linkers that are usually employed exhibit good stability; however, acid‐stable alternatives, such as photolabile or safety‐catch linkers, can be considered if necessary. The latter are stable under various conditions, but become cleavable upon chemical activation [42]. Finally, when establishing new LSF methods, the conditions for peptide cleavage from the resin must also be taken into account. The use of strong acids and nucleophilic additives, such as triisopropylsilane or thiols, can induce undesired side reactions.

### 2.2 Orthogonal Protecting Group Strategies

In addition to the points outlined above, protecting group strategies must be carefully considered when planning an LSF to selectively target the reactive site within the peptide sequence to be modified. In LSF, amino acid building blocks bearing the required reactive groups are incorporated into the peptide sequence during SPPS. In most cases, an orthogonal side chain protecting group strategy is required to selectively address these residues, enabling the reactive groups to be deprotected while the side chains of the other amino acids in the peptide sequence remain protected. In standard Fmoc/*t*‐Bu protection group strategies, the functional groups of the amino acid side chains are protected using acid‐sensitive groups. Protecting groups that are typically considered to be orthogonal to standard side chain protecting groups in Fmoc/*t*‐Bu SPPS include allyl protecting groups, such as allyl esters for carboxylic acids or the allyloxycarbonyl (Alloc) group for amino groups, as well as highly acid‐sensitive groups, such as 4‐methyltrityl (Mtt), monomethoxytrityl (Mmt) or dimethoxytrityl (Dmt), which are used for the orthogonal protection of alcohols, thiols and amines [43, 44, 45, 46, 47, 48]. Allyl‐based protecting groups are cleaved under metal catalysis using Pd(0) and nucleophilic additives such as phenylsilane [43, 44, 45]. Deprotection generally proceeds smoothly; however, residual palladium catalyst on the resin can induce side reactions in subsequent steps. Conversely, highly acid‐sensitive groups are usually removed using a solution of 1% trifluoroacetic acid in dichloromethane [46, 47, 48]. While most protecting groups of the standard amino acid building blocks are stable under these conditions, partial deprotection of trityl‐protected cysteine has been observed [48]. Alternatively, photolabile protecting groups are available for most functional groups [49, 50]. Although deprotection occurs under light irradiation and neutral conditions, rendering them fully orthogonal to the Fmoc/*t*‐Bu protecting group strategy, low deprotection efficiencies and side reactions caused by photoproducts can sometimes be problematic. A variety of other protective groups are available that are compatible with the general conditions of SPPS. Examples include hydrazine‐sensitive groups such as 4‐(*N*‐[1‐(4,4‐dimethyl‐2,6‐dioxocyclohexylidene)‐3‐methylbutyl]amino)benzyl (Dmab) for carboxylic acids, 1‐(4,4‐dimethyl‐2,6‐dioxocyclohex‐1‐ylidene)‐3‐methylbutyl (ivDde) for amino groups and silyl protecting groups for alcohols [51]. The latter are removed using tetrabutylammonium fluoride.

The wide variety of orthogonal protecting groups enables stepwise LSF at multiple sites directly on the resin, provided a suitable protecting group strategy is employed. While all canonical amino acids with Fmoc and standard side chain protection are commercially available in quantities of several tons, this is not the case for other side chain protecting groups, which must first be synthesized. Ideally, a selective reaction on unprotected peptides would be employed for reasons of chemical elegance and atom economy, but this is only possible for a handful of reactions, such as click reactions [52].

## 3 On‐Resin Nucleophilic Substitution

### 3.1 Resin‐Bound Peptide as Nucleophile

Nucleophilic groups are naturally present in peptides and proteins. These include amino groups in lysine, hydroxyl groups in serine and threonine, phenol groups in tyrosine and thionucleophiles in methionine and cysteine. Nucleophilic substitution is therefore a popular and powerful method for functionalizing peptides at a late stage of synthesis.

**FIGURE 2:**
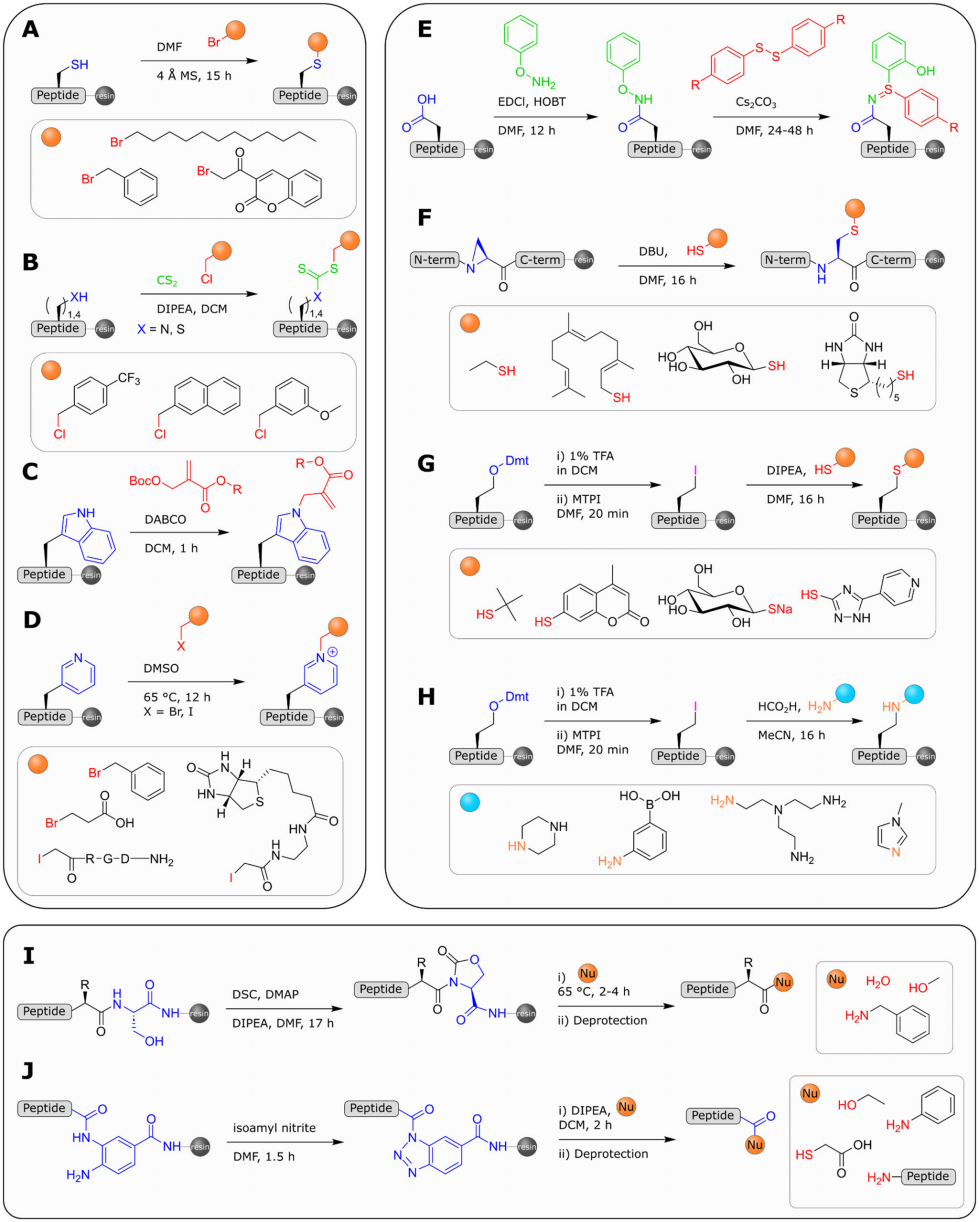
On‐resin nucleophilic substitution of peptides with selected examples from the substrate scope. (A) Modification of cysteine‐containing peptides with bromoalkanes; (B) tri‐ and dithionate formation with benzyl chlorides and cysteine or lysine; (C) amine‐catalyzed tryptophan allylation; (D) alkylation of pyridyl side chains; (E) sulfilimine formation of *N*‐phenoxypeptidylamines; (F) nucleophilic aziridine‐opening; (G) nucleophilic substitution in iodohomoalanine residues with thiols; (H) amination of iodohomoalanine residues; (I) *C*‐terminal peptide modification by substitution of a cyclic urethane linker; (J) nucleophilic *C*‐terminal peptide modification after on‐resin benzotriazole formation.

Due to their high reactivity, strategies targeting cysteine side chains are the most frequently described. Many methods that allow peptide modification in solution have been reported, with maleimide conjugation being the most popular one [53, 54, 55]. On‐resin approaches offer good control over site selectivity due to the possibility of using orthogonal protecting group strategies (Chapter 2.2). In this context, De Luca et al. modified resin‐bound peptides containing cysteine residues via nucleophilic substitution of haloalkanes in an S_N_2 reaction in DMF with molecular sieves; the scope of the electrophiles also included lipids and fluorophores (Figure 2A) [56]. Likewise, Shinde et al. developed a method for trithioate formation starting from cysteine‐containing peptides. The reaction involves a nucleophilic addition to carbon disulfide, followed by a nucleophilic substitution on benzyl chloride derivatives (Figure 2B) [57]. In addition to cysteine, the lysine side chain or the *N*‐terminus of the peptide have been shown to also act as nucleophiles in this reaction, in which case dithioates are formed instead.

In general, *N*‐nucleophiles have frequently been targeted. Among these, amide formation reactions on lysine side chains and its lower homologs are particularly well‐established and popular [58]. Other *N*‐nucleophiles have been studied less frequently. One rare example is the DABCO‐catalyzed *N*‐allylation of the indole ring in tryptophan residues, as described by Liu et al. (Figure 2C) [59]. In addition to the nucleophiles that occur naturally in peptides, non‐canonical amino acids have also been employed in nucleophilic substitutions on resin. The group of Bandyopadhyay demonstrated *N*‐alkylation on pyridyl‐alanine (Figure 2D) [60]. The scope of substrates included complex electrophiles such as biotin and RGD peptide iodoacetamides. While the reaction worked well with halogen acetamides and benzyl halides, lower yields were obtained with less electrophilic haloalkanes. The amide of the peptide backbone can also be modified by nucleophilic substitution, for instance, using methyl iodide and sodium hydride as a base as shown by Ostresh et al. [20]. However, due to the harsh reaction conditions, only amino acids with aliphatic side chains and phenylalanine were tested and peptide epimerization was observed. White et al. reported on the *N*‐methylation of cyclic peptides using methyl iodide and lithium *tert*‐butoxide as a base, a method compatible with amino acid side chains that are protected as ethers or esters [61]. The *N*‐methylated products were obtained with high selectivity with the pattern of methylation being influenced by the stereochemistry and geometry of the peptide backbone, as well as by the choice of solvent.

The modification of hydroxyl groups is a common method of introducing post‐translational modifications. Examples include the introduction of carbohydrates via Schmidt glycosylation, or phosphorylation using dibenzyl phosphochloridate or di‐*tert*‐butyl‐*N*,*N*‐diethylphosphoramidite [10, 11, 12, 13]. *N^ε^*‐phosphorylation of lysine can also be achieved via a Staudinger phosphite reaction, as demonstrated by Hackenberger and co‐workers [62]. Modification of phenol groups via on‐resin tyrosine sulfation was demonstrated by Payne and co‐workers [63].

### 3.2 Resin‐Bound Peptide as Electrophile

As mentioned above, electrophilic functional groups do not occur naturally in peptides and proteins. However, it has been demonstrated that electrophilic entities can be introduced into peptides at a late stage of synthesis, after which they are subjected to nucleophilic substitution reactions. One example is the sulfimidation of *N*‐phenoxypeptidylamines, as demonstrated by He et al. [64]. This process involves the coupling of *O*‐phenylhydroxylamine to a carboxylic group in aspartate or glutamate, followed by a reaction with diaryldisulfides. The group demonstrated the broad scope of substrates, including complex modifications such as biotin, flurbiprofen, and coumarin (Figure 2E).

Galonić et al. reported a method of incorporating electrophilic groups into peptides that enables peptide modification without the formation of large linkers. More specifically, they incorporated aziridine‐2‐carboxylic acid into peptides, which were then reacted with thiols to form cysteine‐derived thioethers (Figure 2F) [16, 17]. Additional precautions were required to ensure the compatibility of the aziridine group with SPPS, such as using 1% 1,8‐diazabicyclo[5.4.0]undec‐7‐ene (DBU) in DMF for Fmoc deprotection, since the typical conditions of 20% piperidine in DMF led to aziridine ring opening and deacylation. The nucleophilic ring opening of the aziridine was controlled by adding catalytic amounts of DBU, enabling the reaction to proceed with high regioselectivity and yielding cysteine‐derived thioethers.

Another example of nucleophilic substitution on resin‐bound peptide electrophiles is the iodination‐substitution approach described by the Thomas group (Figure 2G) [46]. In this approach, homoserine is incorporated into the desired peptide sequence using SPPS, with the side chain protected by the highly acid‐sensitive Dmt group. Following selective deprotection, homoserine is converted to iodohomoalanine in an Appel‐type reaction using the iodination reagent methyltriphenoxyphosphonium iodide (MTPI). The iodohomoalanine residue has been shown to react with various *S*‐nucleophiles, including the sterically hindered *tert*‐butyl mercaptan, mercaptocoumarin, and thioglucose. The use of amines was initially hampered by the competing elimination reaction. However, by changing the solvent from DMF to acetonitrile and adding formic acid, this side reaction could be minimized (Figure 2H) [36]. The procedure was feasible with various *N*‐nucleophiles, including primary and secondary amines, anilines and *N*‐heterocycles. Interestingly, substrates with multiple amino groups reacted only via one nucleophilic site, with no overalkylation or peptide cross‐linking observed. The iodination‐substitution approach was demonstrated on peptides of varying lengths, including a WW domain with 34 amino acids.

Incidentally, there are reports of serine halogenation; however, the resulting haloalanine is highly susceptible to side reactions, including elimination, which can result in the formation of either dehydroalanine or 2‐oxazoline, as demonstrated by Meldal et al., whereby the formation of 2‐oxazolines represents an interesting example of an on‐resin backbone modification [18, 19]. Dehydroalanine can react as a Michael acceptor and be obtained on the solid phase via oxidative elimination of Se‐phenylselenocysteine with hydrogen peroxide. This process was demonstrated by van der Donk and co‐workers, who subsequently synthesized glycopeptides via Michael addition [22]. Nonetheless, the use of dehydroalanine as an electrophile has the disadvantage of diastereomer formation. An alternative strategy for introducing an electrophilic group to a resin‐bound peptide is the preparation of allenamide—an orthogonal handle for reacting with thiols – as reported by Brimble and colleagues [65].

### 3.3 *C*‐Terminal Modification

In addition to side chain modifications, on‐resin nucleophilic substitution has been employed to introduce *C*‐terminal modifications. One simple way to achieve this is to prepare a side chain‐anchored peptide with an orthogonally protected *C*‐terminus, for example by incorporating *N^α^*‐allyl esters of glutamic or aspartic acid as *C*‐terminal amino acid building blocks. Using this approach, Ficht et al. introduced *C*‐terminal thioesters by coupling the carboxylic acid with thiols using *N,N'*‐diisopropylcarbodiimide, after orthogonally deprotecting the *C*‐terminus [66]. The Raj group reported *C*‐terminal modification via on‐resin formation of a cyclic urethane (Figure 2I) [67]. First, the *C*‐terminal serine is activated with *N*,*N*'‐disuccinimidyl carbonate (DSC) and converted into a cyclic urethane via nucleophilic attack on the amide backbone. The *C*‐terminus is then modified by nucleophilic substitution. This method can be used to synthesize *C*‐terminal esters, amides and alcohols. However, the scope of the tested nucleophiles was limited to water, methanol, and benzylamine as the publication focused on the production of macrocyclic peptides. More recently, Selvaraj et al. developed a method for the *C*‐terminal modification of peptides on the solid phase, which was inspired by *C*‐terminal thioester synthesis (Figure 2J) [68]. The key step of this approach is the mild conversion of the *N*‐acyl‐benzimidazoline linker to benzotriazole through treatment with isoamyl nitrite. The peptide is released from the resin through reaction with a nucleophile. *C*‐terminal modification was possible using water, alcohols, thiols and amines; even unreactive aniline yielded 66%. Notably, this method has also been applied in head‐to‐tail cyclization and the synthesis of longer peptides using other peptides as nucleophiles. It was also used for the *C*‐terminal addition of aminonitriles to produce macrocyclic thiazole peptides, demonstrating the versatility of this approach [69].

## 4 On‐Resin Carbonyl Chemistry

### 4.1 Reductive Amination

**FIGURE 3:**
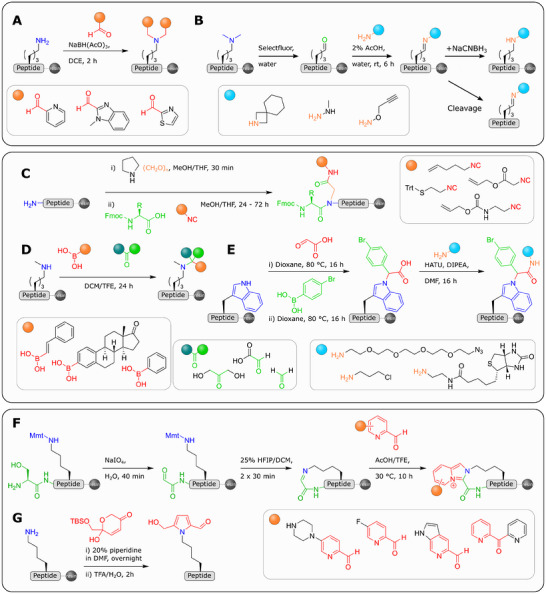
On‐resin modification of peptides through carbonyl chemistry with selected examples from the substrate scope. (A) Lysine modification by reductive amination; (B) conversion of dimethyllysine to allysine followed by imination and reduction; (C) Ugi reaction at the peptide *N*‐terminus; (D) Petasis reaction on methyllysine; (E) Petasis reaction on tryptophan; (F) synthesis of imidazopyridinium macrocyclic peptides; (G) conversion of lysine to pyrraline.

The natural abundance of *N*‐nucleophiles in peptides, whether located at the *N*‐terminus, the tryptophan indole ring or the lysine side chain, allows for carbonyl chemistry based on imine formation. Reductive amination on resin‐bound peptides has been employed in various studies, either utilizing the lysine side chain or the *N*‐terminus as the amine source [70, 71, 72, 73, 74, 75, 76, 77]. Most protocols employ an excess of aldehyde as the carbonyl compound, dissolved in dichloromethane or dichloroethane with acetic acid added. Na(OAc)_3_BH or, in some cases NaCNBH_3_, is subsequently used to reduce the imine to the corresponding amine. Dipyridylamine‐containing peptides synthesized in this way have a wide range of applications (Figure 3A) [75, 76]. An early example by Quinti et al. demonstrated the use of this technique to produce phosphatidylserine‐binding peptide ligands based on the phosphate‐sensitive properties of the Zn(II)‐dipicolylamine complex [70]. Zwicker et al. also applied this concept to produce fluorogenic peptide probes for phosphatidylserine on the cell surface [74]. Further applications from the He group include forming a Mn(I) carbonyl complex with a dipicolylamine‐containing peptide to enable light‐controlled release of CO, and designing an artificial metalloenzyme by mimicking the histidine brace of a lytic polysaccharide monooxygenase by incorporating a dipicolylamine motif into the active site of azurins [75, 76]. Catalytic activity of this minienzyme was characterized by a total turnover number of 253, a turnover rate of 1.61 min^−1^, and a Michaelis–Menten constant of 0.5 mM.

Reductive amination has also been applied with the resin‐bound peptide acting as the carbonyl source and an external amine. Aldehydes do not occur naturally in peptides and must therefore be introduced by either SPPS using unnatural amino acids or LSF. Chakraborty et al. introduced an aldehyde into the peptide side chain by oxidizing hydroxynorleucine on a resin‐bound *N*‐terminal H3 peptide with Dess–Martin periodinane (DMP) [71]. The resulting allysine was then modified by reductive amination. It should be noted that this method has only been demonstrated with one peptide, and that oxidation with DMP is incompatible with tryptophan residues [15]. More recently, Emenike et al. developed a new strategy for accessing allysine by converting dimethyllysine with Selectfluor on water‐swellable resin (Figure 3B) [78]. Allysine was then further modified with amines, hydrazines or hydroxylamines to produce the corresponding imino compounds. These were either isolated directly as iminopeptides in the case of the hydrazone or the oxime, or further reduced with NaCNBH_3_ to produce *N*‐alkylated products.

### 4.2 Multicomponent Reactions

Multicomponent reactions are highly valuable for discovering peptide therapeutics because they allow for a greater degree of diversification in the peptides produced [79]. The Ugi reaction is one such reaction that has proven useful for on‐resin multicomponent backbone *N*‐modification [80]. In this regard, Ricardo et al. reported the incorporation of isocyanides in the peptide backbone in combination with an aminocatalytic transimination of paraformaldehyde. This approach facilitated the use of formaldehyde as carbonyl component to prevent the formation of diastereomeric mixtures. Furthermore, the synthetic versatility of this platform was demonstrated by combining the Ugi reaction with subsequent backbone stapling, including ring‐closing metathesis, macrolactamization, and bis‐alkylation (Figure 3C). The same group also reported an optimized Petasis reaction on the solid phase for peptide modification [14, 81]. Their method involves subjecting methylated lysine side chains or the *N*‐terminus of a peptide to the Petasis reaction with a variety of aryl or vinyl boronic acids, as well as aldehydes or ketones (Figure 3D) [81]. Using a primary amine results in a doubly modified product. Similar to the aminocatalytic transimination used for the Ugi reaction, the introduction of formaldehyde can be elegantly solved through transimination with pyrrolidine, whereby the resulting pyrrolidinium ion transfers the formaldehyde group from paraformaldehyde to the resin‐bound amine. Krajcovicova et al. demonstrated that the Petasis reaction can also be applied to tryptophan [82]. The use of glyoxic acid as an aldehyde source introduces an additional carboxyl group, which can be used for further diversification through, for example, amide coupling (Figure 3E).

### 4.3 *N*‐Heterocyclization

Kodadek and co‐workers developed a method for forming imidazopyridinium macrocyclic peptides. First, the *N*‐terminal serine is oxidized to form an aldehyde, which then forms an imine with lysine. Next, 2‐formyl‐ or 2‐ketopyridines trap the reversible intramolecular imine to form an imidazopyridinium moiety (Figure 3F) [83]. This LSF works with a wide range of 2‐formyl‐ and 2‐ketopyridines, thereby leading to cyclic peptides with enhanced passive membrane permeability. Implementing piperazine‐containing 2‐formylpyridine enabled further modification via amide coupling to introduce fluorophore and biotin labels. The same group also described the formation of 2‐pyridone on resin via I_2_/acetone‐mediated condensation of 3,3‐methoxypropionic acid and amines [84, 85]. Pyrraline is an advanced glycation end‐product associated with retinopathy and nephropathy. To access this amino acid in peptides, Brimble and co‐workers converted lysine in collagen peptides to pyrraline through on‐resin Maillard‐type condensation with dihydropyranone (Figure 3G) [86].

While most examples of carbonyl chemistry used in the on‐resin LSF of peptides involve amines as reaction partners, there is one notable exception: an on‐resin Wittig olefination, which was demonstrated by Masri et al. [87] However, this is discussed in more detail in Chapter 7.2, as this strategy also involves a modification based on a 1,3‐cycloaddition. Generally, carbonyl chemistry is highly compatible with solid‐phase peptide chemistry. All the examples shown are applicable to large, complex peptides.

## 5 On‐Resin Metal Catalyzed Reactions

### 5.1 Cross‐Coupling Reactions

**FIGURE 4:**
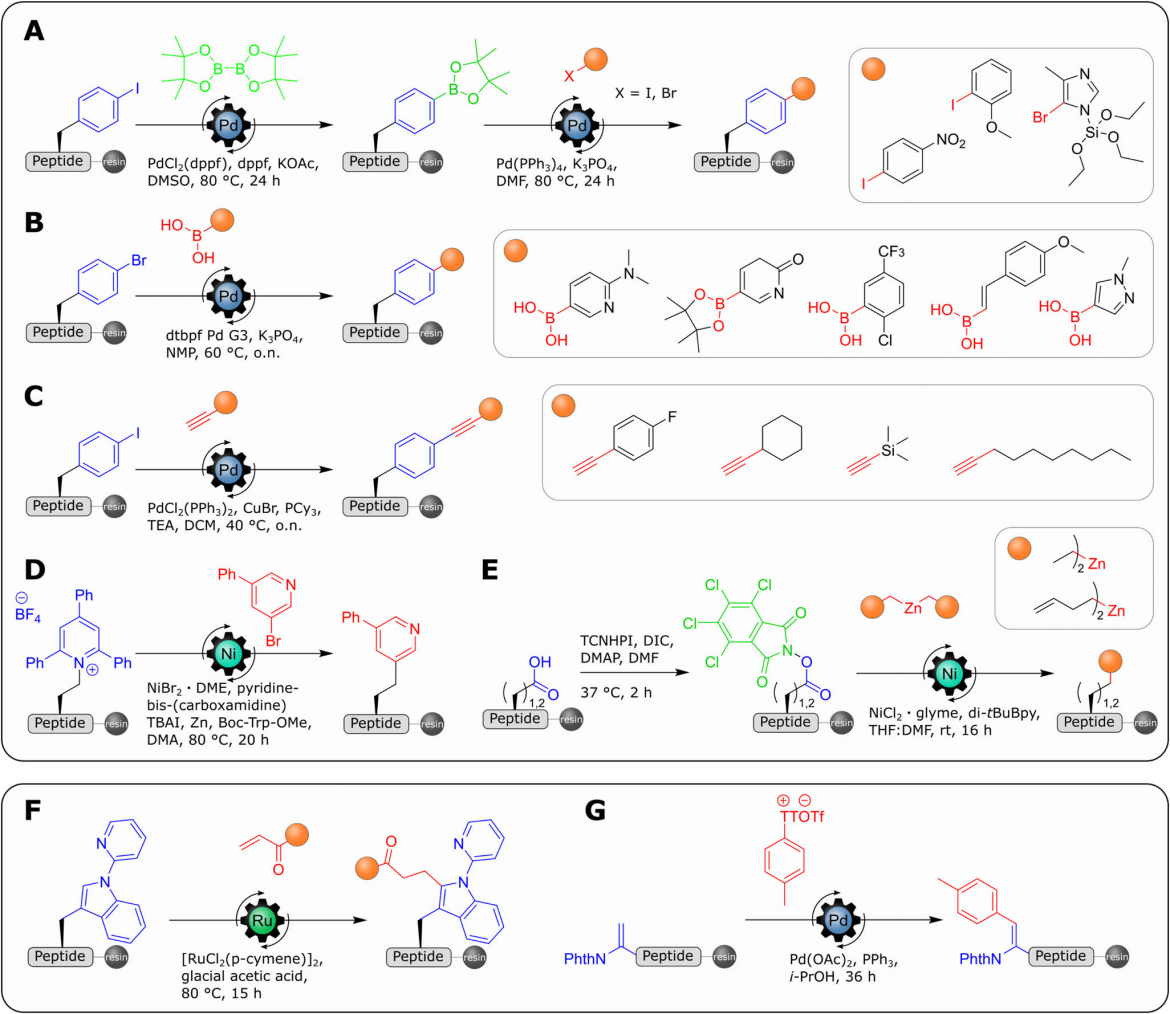
Metal‐catalyzed reactions for on‐resin peptide modification. (A) Miyaura borylation of iodophenylalanine followed by SMC; (B) SMC with resin‐bound bromophenylalanine; (C) on‐resin Sonogashira reaction on iodophenylalanine; (D) deaminative cross‐coupling with pyridinium salts; (E) decarboxylative cross‐coupling with redox‐active esters; (F) C─H activation on resin‐bound tryptophan; (G) C─H activation on resin‐bound *N^α^*‐phthaloyl‐protected dehydroalanine.

The palladium‐catalyzed Suzuki–Miyaura cross‐coupling reaction (SMC) is one of the most widely used methods of forming C─C bonds, primarily because of the mild reaction conditions [88, 89]. It has therefore found its way into peptide chemistry [90]. Various groups, including Haug et al., Doan et al., and Limbach et al., have shown that resin‐bound oligopeptides containing up to seven residues and comprising 4‐iodophenylalanine and 4‐bromophenylalanine can be modified by SMC at elevated temperatures (50°C–90°C) overnight using a variety of boronic acids or boronates. Conversion rates of up to 90% have been achieved [91, 92, 93]. However, a greater variety of aryl halides are commercially available than boronic acid, which has driven the development of palladium‐catalyzed on‐resin borylation strategies for peptides containing 4‐iodophenylalanine and 3‐iodotyrosine as shown by Alfonso et al. (Figure 4A) [94, 95]. The resulting borylated peptides can undergo further modifications, such as the formation of cyclic peptides containing both borylated and halogenated aromatic amino acids. Due to its robustness and ease of execution, SMC is ideal for high‐throughput screening. Shasha et al. used 96‐well filter plates, which were originally developed for solid‐phase extraction, to create libraries of modified peptides [96]. Initially, this setup was employed to evaluate various reaction conditions for the on‐resin functionalization of pentapeptides containing 4‐bromophenylalanine or 5‐bromotryptophan (Figure 4B). The optimized conditions were then employed to synthesize a peptide library via functionalization with aromatic boronic acids or boronates. The SMC is not limited to aromatic amino acids. Jamieson et al. synthesized nonapeptides containing azidohomoalanine and pentynoic acid [97]. Copper‐catalyzed azide‐alkyne cycloaddition, in the presence of NBS as an oxidant, yielded a 5‐iodo‐1,4‐triazole cyclized peptide. This peptide was subsequently modified by SMC with aromatic pinacol boronates with very good to excellent conversion.

The Sonogashira reaction is a common method of forming C─C bonds in organic synthesis, involving the coupling of terminal alkynes and aryl halides in the presence of palladium and copper catalysts [98, 99]. In an example published by Doan et al., octapeptides containing 4‐iodophenylalanine were diversified via Sonogashira coupling with various alkynes, achieving good to excellent conversion (Figure 4C) [100]. Doebelin et al. achieved peptide diversification by coupling 4‐iodobenzoic acid to the *N*‐terminal position of a peptide which is then functionalized by Sonogashira reaction [101]. In addition to using artificial aromatic amino acid building blocks, on‐resin cross‐coupling reactions on canonical aromatic amino acids were also successfully carried out. One example was presented by the Chen group, who conducted copper‐catalyzed on‐resin heteroatom‐arylation on tryptophan, tyrosine and histidine side chains with various aryl diiodides for the purpose of peptide stapling [102].

The application of cross‐coupling reactions for on‐resin peptide LSF is not restricted to peptides containing aromatic amino acids as reaction handles. Twitty et al. showed that treatment of basic amino acids, such as lysine, ornithine, 2,4‐diaminobutyric acid and 2,3‐diaminopropanoic acid, with pyrilium salts converts the amino group of the side chain into a pyridinium group. This pyridinium group is susceptible to cross‐coupling with aryl bromides using a nickel catalyst (Figure 4D) [103]. The reaction was initially optimized for the modification of protected amino acids in solution, but it has also been demonstrated to be effective on the solid phase: a linear pentapeptide and a cyclic decapeptide were successfully modified, both containing mostly aliphatic and aromatic amino acids. The Baran group converted carboxyl groups on resin‐bound peptides, such as glutamic acid and aspartic acid, into redox active esters using the coupling reagent tetrachloro‐*N*‐hydroxyphthalimide tetramethyluronium hexafluorophosphate (CITU). The active esters then react with alkyl zinc reagents under nickel catalysis to form a C─C bond, releasing CO_2_ (Figure 4E) [104, 105]. The reaction was demonstrated on several resin‐bound bioactive peptides, including spinorphin, rubiscolin‐6 and β‐neoendorphin.

### 5.2 C─H Activation Reactions

Although the C─H activation of peptides in solution is well understood and extensively reported [106, 107, 108, 109, 110, 111], its application to resin‐bound peptides is rare. Ackermann and co‐workers demonstrated the modification of tryptophan residues through C─H activation, whereby the indole nitrogen is protected by a 2‐pyridyl group, which serves as a directing group for the ruthenium catalyst employed in this reaction (Figure 4F) [112]. Nona‐ and decapeptides containing mostly aliphatic amino acids were functionalized on resin, with arginine and glutamine residues also being tolerated. Furthermore, the peptide backbone has been shown to function as a directing group in C─H activation. Resin‐bound tripeptides containing an *N^α^*‐phthaloyl‐protected dehydroalanine residue have been shown to undergo modification with arylthianthrenium salts under palladium catalysis, producing dehydrophenylalanine analogs (Figure 4G) [113]. In this case, Dha acts as a nucleophile.

### 5.3 Other Metal‐Catalyzed Reactions

**FIGURE 5:**
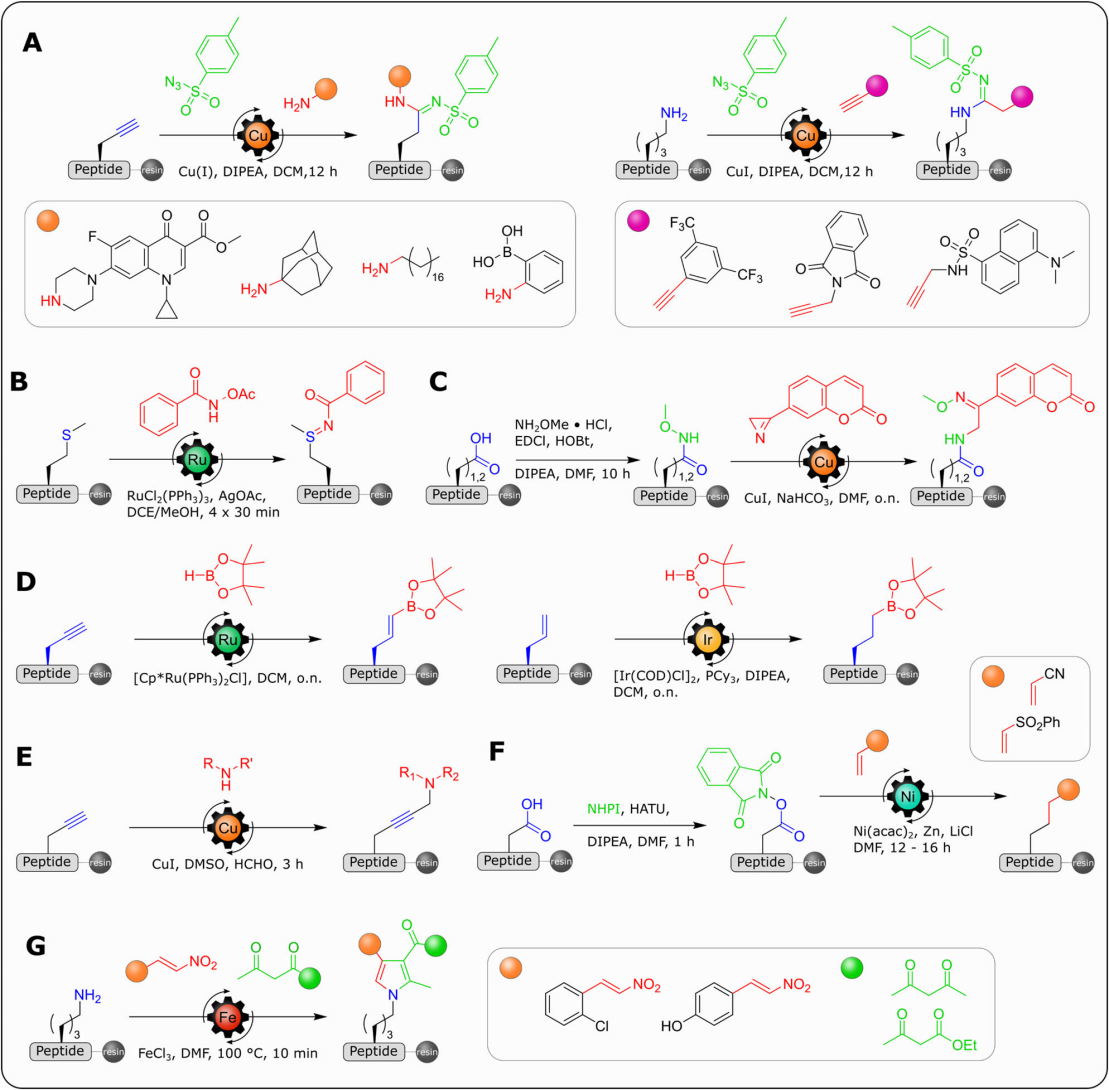
Metal‐catalyzed reactions for on‐resin peptide modification. (A) Ketenimine multicomponent reaction on resin‐bound propargylglycine or lysine; (B) methionine‐selective sulfimidation; (C) on‐resin oxime‐ether formation followed by reaction with 2*H*‐azirines; (D) regioselective hydroboration on resin‐bound propargylglycine and allylglycine; (E) A^3^‐coupling on alkyne‐containing peptides; (F) decarboxylative Giese‐type reaction; (G) lysine‐based pyrrole formation.

The most common copper‐catalyzed LSF of peptides on the resin is the azide‐alkyne cycloaddition. This well‐established reaction has been discussed in detail previously, so it will not be covered in this review [114]. A copper‐catalyzed reaction involving terminal alkynes, sulfonyl azides and amine nucleophiles results in the formation of amidines (Figure 5A) [115]. This reaction, described by the Kiran group, was used to either modify propargyl glycine‐containing dipeptides with a wide scope of aliphatic and aromatic amines or, conversely, lysine side chains with alkyne substrates. Furthermore, it has been demonstrated that the reaction can be used in the synthesis of cyclic tripeptides, in which the reaction occurs between a lysine residue and an alkyne‐containing amino acid. He et al. reported a ruthenium‐catalyzed sulfimidation of methionine residues using *N*‐acetoxyamides as substrates (Figure 5B) [116]. This method has proven suitable for side chain modification and peptide cyclization. Pentapeptides containing a methionine residue and an allyl‐protected glutamate residue can be cyclized using the ruthenium catalyst [RuCl_2_(PPh_3_)_3_]. Prior to cyclization, the allyl‐protected glutamate residue is selectively transformed into the required *N*‐acetoxyamide substrate via allyl deprotection followed by *O*‐acetylhydroxylamine coupling.

The Cu(I)‐catalyzed reaction between *N*‐methoxyamides and 2*H*‐azirines results in the formation of oxime ethers. This reaction has been used to introduce fluorescent labels to resin‐bound peptides (see Figure 5C) [117]. Liu et al. incorporated a glutamate building block during SPPS that was orthogonally protected as an allyl ester in the side chain. This was then coupled with methoxyamine after palladium‐mediated selective deprotection, resulting in the formation of the corresponding methoxyamide. Subsequent reaction with 2*H*‐azirine‐containing coumarin gave the functionalized pentapeptide.

Propargylglycine has proven to be a useful and cost‐effective building block for enabling the LSF of peptides on the solid phase. For example, propargylglycine has been used as a reactive handle in on‐resin ruthenium‐catalyzed hydroboration, which results in the formation of vinylboronic acids (Figure 5D) [38]. Werner et al. used [Cp*Ru(PPh_3_)_2_Cl] as a catalyst and pinacol borane as a substrate, demonstrating high selectivity in the formation of (*E*)‐vinylboronic acids. While a more air‐stable iridium catalyst produces a mixture of regioisomers in the hydroboration of propargylglycine‐modified resins, it is highly effective in the on‐resin hydroboration of allylglycine‐containing peptides, resulting in the respective aliphatic boronic acids. The group demonstrated the general applicability of this approach to various boronates and peptides of up to 58 amino acids in length, achieving good to excellent yields in the reaction. Notably, peptide boronic acids are of great interest in the development of new peptide therapeutics, since these compounds are known to form reversible covalent bonds with naturally occurring diols, such as carbohydrates [118]. Additionally, vinylboronic acids are valuable starting materials for further peptide modification, e.g., through SMC or Petasis reaction.

The reaction between a terminal alkyne, an aldehyde and an amine to form propargylamines, known as A^3^ coupling [119], has also found its way into on‐resin peptide LSF (Figure 5E). Lubell and co‐workers used aza‐propargylglycine building blocks, secondary amines and formaldehyde that reacted under copper catalysis to form aza‐lysine analog‐containing hexapeptides [120, 121]. The reaction is compatible with several secondary amines, including sterically hindered side chains, such as isopropyl or cyclohexyl residues. Cyclic azapeptides are formed in a copper‐catalyzed reaction with formaldehyde when the aza‐propargylglycine is positioned at the *N*‐terminus and the *N*‐alkylated lysine at the *C*‐terminus of the peptide sequence [122].

Similar to the aforementioned decarboxylative cross‐coupling reaction, the Baran group used redox‐active esters in a nickel‐catalyzed Giese‐type reaction on the resin (Figure 5F) [123]. In the Giese reaction, alkyl radicals react with Michael acceptors in a 1,4‐addition. As an alternative to light, these radicals can be generated by nickel‐catalyzed decarboxylation of *N*‐hydroxyphthalimide active esters. This variant of the Giese reaction was employed to functionalize carboxylic groups in resin‐bound tripeptides, including glutamate side chains and the free *C*‐terminus. It was also demonstrated that the amino groups in the lysine side chains could be selectively modified with acrylic acid followed by further conversion to cross‐linked peptides by stapling with *N*‐hydroxyphthalimide active ester modified amino acids. Although radicals are formed during the reaction, tyrosine was tolerated. Other sensitive amino acids were not tested.

The Albericio group employed a microwave‐assisted multicomponent condensation reaction involving a β‐nitrostyrene, a 1,3‐dicarbonyl compound and a free lysine side chain to modify resin‐bound peptides with pyrroles (Figure 5G) [124]. The reaction is catalyzed by FeCl_3_ as a Lewis acid; however, other Lewis acids, such as zinc and aluminium salts, have been shown not to catalyze the reaction. The scope was demonstrated on a lysine‐containing tripeptide. The reaction produces high‐purity crude products and has been shown to be versatile, enabling a variety of pyrroles to be introduced into peptides.

## 6 On‐Resin Redox Reactions

**FIGURE 6:**
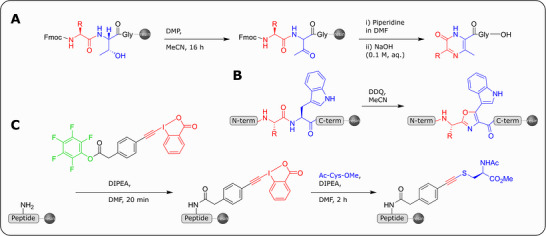
Redox reactions on resin‐bound peptides. (A) Oxidation of threonine side chains followed by pyrazine formation; (B) tryptophan‐selective oxazole formation; (C) thioalkyne formation with ethinylbenziodoxolones.

So far, redox reactions have rarely been used for on‐resin peptide modification. This is most likely due to the risk of side reactions, such as tryptophan, cysteine and methionine oxidation. This was observed during Dess–Martin periodinane‐mediated oxidation of threonine side chains, as reported by the Meldal group in their studies on pyrazine formation on the solid phase (Figure 6A) [15]. The on‐resin oxidation of hydroxynorleucine in an *N*‐terminal H3 peptide, as mentioned previously, demonstrates that DMP‐mediated oxidation can be applied to more complex peptides [71]. The Qvortrup group reported a selective oxidative backbone cyclization involving the formation of an oxazole between the backbone and the indole side chain of tryptophan, using 2,3‐dichloro‐5,6‐cyano‐1,4‐benzoquinone (DDQ) as the oxidizing agent (Figure 6B) [125]. The cyclodehydration results in a fully conjugated indolyl‐oxazole that shows blue fluorescence. It has also been demonstrated that the method is applicable to complex peptides such as GLP‐1. The research conducted by the Waser group on hypervalent iodine reagents clearly demonstrates the benefits of redox reactions in bioorganic chemistry [126]. Ethinylbenziodoxolones react with thiols, such as cysteine, to form thioalkynes (Figure 6C). During peptide cleavage from the resin using trifluoroacetic acid, the thioalkyne is converted into a thioester. However, when synthesized on a trityl resin, cleaving the peptide with hexafluoroisopropanol prevents this conversion, leaving the thioalkyne moiety intact and available for a ruthenium‐catalyzed click reaction with azides.

Redox reactions hold great potential for modifying peptides on the solid phase. We anticipate that more strategies based on this chemistry will emerge in the future. One recent development in this area is the use of photoredox reactions, which will be discussed in detail in Chapter 8.

## 7 On‐Resin Pericyclic Reactions

### 7.1 Inverse Electron Demand Diels–Alder Reaction

**FIGURE 7:**
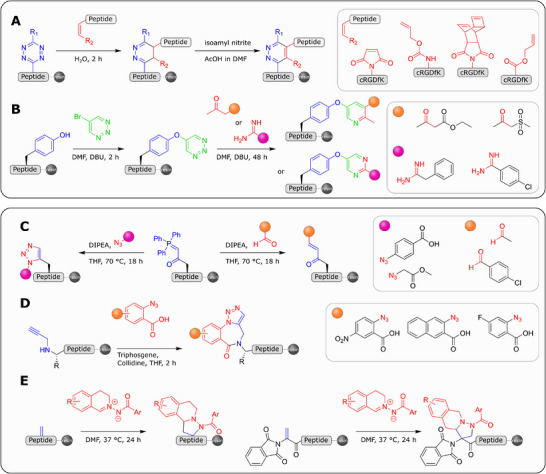
On‐resin pericyclic reactions on resin‐bound peptides. (A) IEDDA with resin‐bound peptide tetrazines; (B) tyrosine triazine ligation strategy; (C) phosphoranylidene‐based cycloaddition with azides followed by Wittig reaction with aldehydes; (D) *N*‐terminal triazolobenzodiazepine formation; (E) on‐resin cycloaddition on Dha residues with azomethins.

Pericyclic reactions are commonly used in bioorganic chemistry for labeling purposes owing to their biorthogonal nature. A prominent example is the inverse electron demand Diels–Alder reaction (IEDDA), which uses electron‐deficient tetrazine compounds as dienes and electron‐rich dienophiles [127]. While this reaction can be performed post‐synthetically in water, eliminating the need to protect other peptide functional groups, the solid phase offers advantages such as avoiding solubility and stability issues of the hydrophobic tetrazine and simplifying purification steps. For this reason, Pagel et al. incorporated a tetrazine into a peptide on resin to attach RGD peptides via IEDDA using four types of dienophiles, including Reppe dienophiles, maleimides, alloc and allyl moieties [128]. The respective conjugates were either cleaved directly from the resin or further oxidized with isoamyl nitrite to yield pyridazines (Figure 7A).

IEDDA can also be used for peptide diversification, as demonstrated by Zuo et al. in their tyrosine‐triazine ligation (Figure 7B) [129]. First, a 1,2,3‐triazine was introduced into a tyrosine side chain. Next, the triazine was modified by IEDDA using various dienophiles, thereby producing either pyridine or pyrimidine moieties. The on‐resin approach allows for parallelization, which was performed in a microplate setup to generate a library of 384 compounds for screening amphiphilic antibacterial peptides.

### 7.2 1,3‐Dipolar Cycloaddition

Masri et al. developed an LSF approach based on the derivatization of a readily accessible phosphoranylidene amino acid from aspartate (Figure 7C) [87]. The resin can be modified by either Wittig olefination with aldehydes or metal‐free 1,3‐dipolar cycloaddition with azides, in order to selectively obtain the 1,5‐triazole. In both cases, the resin is heated in THF with DIPEA for 18 h at 70°C. The compatibility of the unnatural phosphoranylidene amino acid with the standard conditions of SPPS (Fmoc deprotection, amino acid coupling, *N*‐terminal acetylation) was demonstrated. Although the largest peptide in the scope was only a hexamer, it included serine, glutamate, glutamine, and tyrosine, giving it a relatively high level of complexity.

Kamble et al. employed metal‐free on‐resin triazole formation for the *N*‐terminal generation of triazolobenzodiazepines, a notable heterocycle with therapeutic potential (Figure 7D) [130]. The propargyl group is introduced at the *N*‐terminus of the peptide through a Mitsunobu reaction using propargyl alcohol. Subsequent formation of the amide bond with *ortho*‐azidobenzoic acid derivatives and azide‐alkyne cycloaddition proceed without the use of a copper catalyst. This reaction has also been demonstrated using lysine side chains as the amine component.

Dha has also proven to be a valuable building block in the modification of peptides via 1,3‐dipolar cycloaddition. Bao et al. exploited its unique electronic properties, which arise from the electronic properties of the adjacent π‐donating nitrogen and electron‐withdrawing carbonyl group, to introduce *C*,*N*‐cyclic azomethins (Figure 7E) [131]. The regioselectivity was controlled via the *N*‐terminal protecting group of Dha. *N*‐bi‐protected Dha undergoes a 1,3‐cycloaddition with standard electron demand, whereas Fmoc‐protected Dha undergoes a 1,3‐cycloaddition with inverse electron demand. This enables control over the formation of different isomers.

## 8 On‐Resin Photocatalysis

### 8.1 Decarboxylative C─C Bond Formation

**FIGURE 8:**
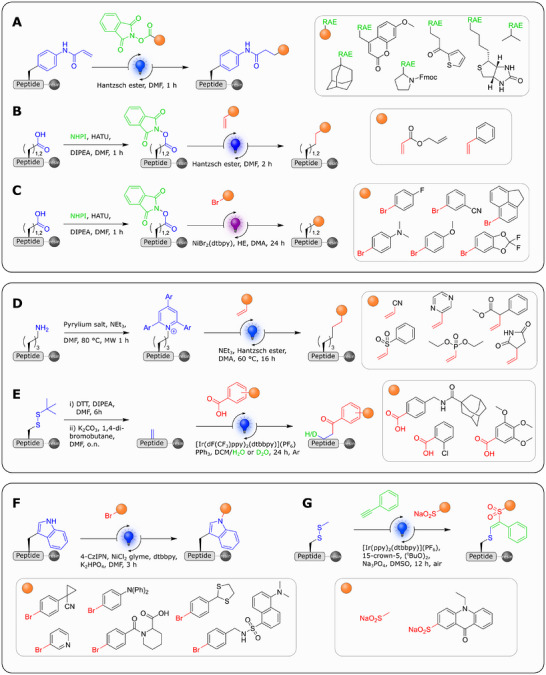
Photocatalyzed peptide modifications on the solid phase. (A) Hydroalkylation of resin‐bound Giese acceptors with redox‐active NHPI esters; (B) Giese‐type reaction with resin‐bound redox‐active NHPI esters; (C) decarboxylative arylation with resin‐bound redox‐active NHPI esters; (D) deaminative C─C bond formation with resin‐bound Katritzky salts; (E) deoxygenative radical addition with resin‐bound dehydroalanine; (F) *N*‐arylation of resin‐bound tryptophan; (G) three‐component atom‐transfer radical addition with resin‐bound disulfide‐protected cysteine.

Photocatalyzed reactions have become increasingly important in organic chemistry, subsequently extending to the solid phase [132]. The Molander group were the first to demonstrate the compatibility of photocatalysis with peptide modification on solid supports, including redox‐sensitive amino acids tryptophan, histidine, cysteine, and methionine, as evidenced by decarboxylative hydroalkylation of resin‐bound Giese acceptors catalyzed by iridium‐based photocatalysts or Hantzsch esters (Figure 8A) [133]. The same group then adopted a different approach, forming redox‐active *N*‐hydroxyphthalimide (NHPI) esters with aspartic and glutamic acids on resin‐bound peptides [134]. This resulted in the formation of an electron donor–acceptor complex with a Hantzsch ester, producing alanyl and homoalanyl radicals via photoinduced single‐electron transfer (Figure 8B). These radicals then reacted with either allyl acrylate or styrene, resulting in the formation of homologs of either glutamic acid or phenylalanine. The ‘t Hart group demonstrated the decarboxylative arylation of redox‐active NHPI esters of aspartic and glutamic acids on resin‐bound peptides using a variety of aryl bromides, yielding phenylalanine and homophenylalanine derivatives (Figure 8C) [37]. Unlike the other reactions shown in this chapter that use blue light, the Hantzsch ester photocatalytic reaction involved the use of a nickel catalyst and a purple LED.

### 8.2 Photocatalyzed Deamination and Deoxygenation

Katritzky salts have been shown to be valuable starting materials for C─C bond formation. They can be generated from lysine or shorter homologs by converting the amino group with pyrylium salts (Figure 8D) [135]. Openy et al. used the resulting Katritzky salts in a photochemical deaminative alkylation, in which an electron donor–acceptor complex forms between a Hantzsch ester and the salt. When irradiated with blue light, the C─N bond undergoes homolytic cleavage to generate a carbon radical, which then reacts with a Michael acceptor. The scope includes a large variety of Michael acceptors and the method proved to be compatible with 15 amino acid residue peptides.

A classical Michael acceptor that can be incorporated into peptides is the amino acid Dha, which reacts with radicals in an addition reaction (Figure 8E) [136]. However, since Michael acceptors can also react with nucleophiles such as piperidine, Bao et al. used disulfide‐protected cysteine as a Dha precursor. Selective deprotection, followed by elimination with potassium carbonate and 1,4‐dibromobutane, produced Dha, which then reacted with carbonyl radicals. These radicals were generated by reacting an aryl carboxylic acid with triphenylphosphine in the presence of an iridium photocatalyst and blue light.

### 8.3 Photocatalyzed Heteroatom Functionalization

The selective *N*‐modification of tryptophan can be challenging due to the presence of several other reactive positions in the indole ring. Selective *N*‐arylation on the resin was achieved by Delgado et al. using a nickel‐dtbbpy complex and 1,2,3,5‐tetrakis(carbazol‐9‐yl)‐4,6‐dicyanobenzene (4‐CzIPN) as a photocatalyst, with a variety of aryl bromides serving as substrates, under blue light irradiation (Figure 8F) [137]. This reaction was shown to be compatible with all canonical amino acids bearing the common protecting groups for Fmoc‐SPPS, making it extremely useful for functionalizing bioactive peptides. The authors were able to selectively functionalize linear and disulfide‐cyclized somatostatin, melittin, glucagon, and others.

As previously mentioned, cysteine is a very useful amino acid for LSF. Using an iridium photocatalyst under blue light irradiation, resin‐bound peptides containing a disulfide‐protected cysteine residue were reacted with phenylacetylene and a sulfinate‐containing dye to obtain a redox‐sensitive fluorescent probe (Figure 8G) [138]. This atom‐transfer radical addition reported by Liu et al. yielded various (*E*)‐β‐alkylsulfonylvinyl alkylsulfides. These dyes exhibit blue fluorescence in the oxidized state and green fluorescence in the reduced state. When combined with cell‐penetrating peptides, the redox state of cells can be visualized using fluorescence microscopy.

## 9 Late‐Stage Functionalization and Peptide Drug Development

On‐resin LSF offers great potential for developing peptide drugs. Incorporating non‐canonical amino acids into potential therapeutic peptides can improve their efficacy and pharmacokinetics [7]. The advantages of on‐resin peptide LSF are that non‐canonical amino acids can be introduced in a time‐ and cost‐efficient manner, and in parallel formats. While most of the research highlighted in this review focuses on developing chemical methods for LSF, in some studies the developed approaches are used to improve properties of potential peptide therapeutics and even highlight LSF in the development of peptide drugs in parallel formats.

Strategies that improve the properties of biologically active peptides include peptide cyclization and stapling. Krajcovicova et al. used the Petasis reaction on the tryptophan side chain (Chapter 4.2) to staple the cell adhesion motifs KQAGDV (muscle cell adhesion) and DGEA (α2β1 integrin receptor) [82]. They were then able to demonstrate pre‐organization of the biologically active conformation and improved biostability compared to the linear counterparts. Lubell and colleagues also used stapling via an A^3^ coupling to produce peptidomimetics that modulate the activity of the multifunctional cluster of differentiation 36 (CD36) scavenger receptor [122]. From a small library of stapled peptides, they identified peptide ligands with *IC*_50_ values at submicromolar concentrations. However, peptide diversification was achieved by varying the peptide sequence rather than LSF. An interesting example of how the pharmacokinetics of peptides can be improved through LSF is the introduction of an imidazopyridinium group into the backbone of peptide macrocycles, as described by Bo et al. (Chapter 4.3) [83]. Despite having molecular weights greater than 500 Da, macrocyclic peptides containing the imidazopyridinium group exhibited excellent membrane permeability. Furthermore, the extent of membrane permeability could be adjusted by varying the substituents at the imidazopyridinium modification.

The main potential of on‐resin peptide LSF is its application in peptide diversification in parallel formats to produce peptide libraries. In a small scale, this was demonstrated by Doan et al. who used Sonogashira coupling on resin‐bound peptides [100]. They identified two peptide variants with improved bioactivity from a set of 10 peptide derivatives of pituitary adenylate cyclase‐activating polypeptide (PACAP), a neuroprotective hormone (Chapter 5.1). Shasha et al. demonstrated that high‐throughput experimentation in an on‐resin SMC in a 96‐well plate format can be used to create large peptide libraries for screening for therapeutic potential (Chapter 5.1) [96]. Zuo et al. took this a step further by using tyrosine‐1,2,3‐triazine ligation on resin‐bound peptides – a reaction sequence consisting of electrophilic aromatic substitution and IEDDA – and applying this method to the high‐throughput synthesis of a library of 384 antimicrobial peptides in a 96‐well plate format, thereby identifying a candidate with good activity against Gram‐positive bacteria [129]. This impressive example demonstrates the potential of late‐stage peptide modification in streamlining peptide drug discovery.

## 10 Conclusions

Over the past decade, we have witnessed the evolution of SPPS, which has progressed from simple peptide sequence assembly to enabling the introduction of non‐canonical modifications in the late stages of the synthetic process. Many classic organic reactions can now be performed on the solid support, which makes diversifying peptides simple and cost‐efficient. This opens new possibilities for peptide drug development. Consequently, the potential for late‐stage diversification through the selective modification of amino acid residues in peptide sequences is becoming increasingly recognized. The main advantage of site‐selective peptide modification on the solid phase is the possibility of parallelizing this process, enabling the development of high‐throughput experiments and the straightforward synthesis of peptide libraries. Some of the contributions in this field provide a glimpse of this potential, particularly the examples demonstrating high‐throughput peptide diversification in 96‐well plate formats [96, 129]. Although this development is still in its infancy, we believe that the potential of on‐resin late‐stage peptide diversification will be unleashed in the next years, especially when combined with the computational tools currently being developed to predict the interaction of modified peptides with therapeutic targets [139]. However, much work remains to be done. The range of organic reactions suitable for LSF that are also compatible with a broad scope of peptides needs to be expanded to allow greater structural flexibility in the modifications introduced. This also involves the further development of experimental setups that facilitate the synthesis and characterization of peptides in parallel formats under different conditions, including photochemistry and electrochemistry.

## Author Contributions

**Marius Werner**: writing – original draft, conceptualization, writing – review and editing. **Truc Lam Pham**: writing – original draft, writing – review and editing, conceptualization. **Franziska Thomas**: writing – original draft, writing – review and editing, conceptualization.

## Funding

Dieter Schwarz Foundation, Max‐Planck‐Gesellschaft, Ministerium für Wissenschaft, Forschung und Kunst Baden‐Württemberg, Bundesministerium für Bildung und Forschung, Deutsche Forschungsgemeinschaft, https://doi.org/10.13039/501100013368, Max Planck School Matter to Life, Deutsche Akademie der Naturforscher Leopoldina ‐ Nationale Akademie der Wissenschaften

## Conflicts of Interest

The authors declare no conflicts of interest.

## Supporting information




**Supporting File**: anie72996‐sup‐0001‐SupMat.pdf.
We have added a small guideline of practical considerations as supplementary material. The authors have cited additional references within the Supporting Information [140, 141, 142, 143, 144, 145, 146, 147, 148, 149, 150, 151, 152, 153, 154, 155, 156, 157, 158, 159, 160, 161, 162].

## Data Availability

This is not relevant, since the manuscript is a review.

## References

[1] M. Muttenthaler , G. F. King , D. J. Adams , and P. F. Alewood , “Trends in Peptide Drug Discovery,” Nature Reviews Drug Discovery 20 (2021): 309–325, 10.1038/s41573-020-00135-8. 33536635

[2] M. Erak , K. Bellmann‐Sickert , S. Els‐Heindl , and A. G. Beck‐Sickinger , “Peptide Chemistry Toolbox—Transforming Natural Peptides Into Peptide Therapeutics,” Bioorganic & Medicinal Chemistry 26 (2018): 2759–2765, 10.1016/j.bmc.2018.01.012. 29395804

[3] A. A. Vinogradov , Y. Yin , and H. Suga , “Macrocyclic Peptides as Drug Candidates: Recent Progress and Remaining Challenges,” Journal of the American Chemical Society 141 (2019): 4167–4181, 10.1021/jacs.8b13178. 30768253

[4] P. Barman , S. Joshi , S. Sharma , S. Preet , S. Sharma , and A. Saini , “Strategic Approaches to Improvise Peptide Drugs as Next Generation Therapeutics,” International Journal of Peptide Research and Therapeutics 29 (2023): 61, 10.1007/s10989-023-10524-3. 37251528 PMC10206374

[5] L. Wang , N. Wang , W. Zhang , et al., “Therapeutic Peptides: Current Applications and Future Directions,” Signal Transduction Targeted Therapy 7 (2022): 48, 10.1038/s41392-022-00904-4. 35165272 PMC8844085

[6] L. Moroder and H.‐J. Musiol , “Insulin—From its Discovery to the Industrial Synthesis of Modern Insulin Analogues,” Angewandte Chemie, International Edition 56 (2017): 10656–10669, 10.1002/anie.201702493. 28548452

[7] J. L. Hickey , D. Sindhikara , S. L. Zultanski , and D. M. Schultz , “Beyond 20 in the 21st Century: Prospects and Challenges of Non‐canonical Amino Acids in Peptide Drug Discovery,” ACS Medicinal Chemistry Letters 14 (2023): 557–565, 10.1021/acsmedchemlett.3c00037. 37197469 PMC10184154

[8] C. Lamers , “Overcoming the Shortcomings of Peptide‐Based Therapeutics,” Future Drug Discovery 4 (2022): FDD75, 10.4155/fdd-2022-0005.

[9] A. Isidro‐Llobet , M. Álvarez , and F. Albericio , “Amino Acid‐Protecting Groups,” Chemical Reviews 109 (2009): 2455–2504, 10.1021/cr800323s. 19364121

[10] K. M. Halkes , C. H. Gotfredsen , M. Grøtli , L. P. Miranda , J. Ø. Duus , and M. Meldal , “Solid‐Phase Glycosylation of Peptide Templates and On‐Bead MAS‐NMR Analysis: Perspectives for Glycopeptide Libraries,” Chemistry – A European Journal 7 (2001): 3584, 10.1002/1521-3765(20010817)7:16<3584::AID-CHEM3584>3.0.CO;2-Z. 11560330

[11] K. C. Nicolaou , S. Y. Cho , R. Hughes , et al., “Solid‐ and Solution‐Phase Synthesis of Vancomycin and Vancomycin Analogues With Activity Against Vancomycin‐Resistant Bacteria,” Chemistry – A European Journal 7 (2001): 3798–3823, 10.1002/1521-3765(20010903)7:17<3798::AID-CHEM3798>3.0.CO;2-6. 11575782

[12] L. Otvos Jr, I. Elekes , and V. M.‐Y. Lee , “Solid‐phase Synthesis of Phosphopeptides,” International Journal of Peptide and Protein Research 34 (1989): 129–133. 2807730 10.1111/j.1399-3011.1989.tb01501.x

[13] J. W. Perich , “Efficient Solid Phase Synthesis of Mixed Thr( P )‐, Ser( P )‐ and Tyr( P )‐containing Phosphopeptides by “global”“phosphite‐triester” Phosphorylation*,” International Journal of Peptide and Protein Research 40 (1992): 134–140, 10.1111/j.1399-3011.1992.tb01461.x. 1280250

[14] S. R. Klopfenstein , J. J. Chen , A. Golebiowski , M. Li , S. X. Peng , and X. Shao , “A Practical Synthesis of Peptide Mimetics via the Solid‐phase Petasis Reaction,” Tetrahedron Letters 41 (2000): 4835–4839, 10.1016/S0040-4039(00)00668-7.

[15] C. Christensen , C. W. Tornøe , and M. Meldal , “Pyrazines on Solid Support From Peptides by Periodinane Oxidation of Threonine Side‐Chains. A Quantitative Chemical Transformation (QCT) for Combinatorial Chemistry,” Qsar & Combinatorial Science 23 (2004): 109–116, 10.1002/qsar.200320009.

[16] D. P. Galonić , W. A. van der Donk , and D. Y. Gin , “Site‐Selective Conjugation of Thiols With Aziridine‐2‐Carboxylic Acid‐Containing Peptides,” Journal of the American Chemical Society 126 (2004): 12712–12713. 15469231 10.1021/ja046793x

[17] D. P. Galonić , N. D. Ide , W. A. van der Donk , and D. Y. Gin , “Aziridine‐2‐carboxylic Acid‐Containing Peptides: Application to Solution‐ and Solid‐Phase Convergent Site‐Selective Peptide Modification,” Journal of the American Chemical Society 127 (2005): 7359–7369. 15898784 10.1021/ja050304r

[18] J. M. Benito and M. Meldal , “Bicyclic Organo‐Peptides as Selective Carbohydrate Receptors: Design, Solid‐phase Synthesis, and On‐bead Binding Capability,” Qsar & Combinatorial Science 23 (2004): 117–129, 10.1002/qsar.200320011.

[19] J. M. Benito , C. A. Christensen , and M. Meldal , “Versatile Solid‐Phase Synthesis of Peptide‐Derived 2‐Oxazolines. Application in the Synthesis of Ligands for Asymmetric Catalysis,” Organic Letters 7 (2005): 581–584, 10.1021/ol047675h. 15704899

[20] J. M. Ostresh , G. M. Husar , S. E. Blondelle , B. Dörner , P. A. Weber , and R. A. Houghten , “"Libraries From libraries": Chemical Transformation of Combinatorial Libraries to Extend the Range and Repertoire of Chemical Diversity,” Pnas 91 (1994): 11138–11142, 10.1073/pnas.91.23.11138. 7972024 PMC45182

[21] D. H. Coy , S. J. Hocart , and Y. Sasaki , “Solid Phase Reductive Alkylation Techniques in Analogue Peptide Bond and Sidechain Modification,” Tetrahedron 44 (1988): 835–841, 10.1016/S0040-4020(01)86120-2.

[22] Y. Zhu and W. A. van der Donk , “Convergent Synthesis of Peptide Conjugates Using Dehydroalanines for Chemoselective Ligations,” Organic Letters 3 (2001): 1189–1192, 10.1021/ol015648a. 11348191

[23] J. Shi , T. Sun , and M. Yang , “Site‐selective Editing of Peptides via Backbone Modification,” Organic Chemistry Frontiers 11 (2024): 1623–1640, 10.1039/D3QO01980B.

[24] A. Boto , C. C. González , D. Hernández , I. Romero‐Estudillo , and C. J. Saavedra , “Site‐selective Modification of Peptide Backbones,” Organic Chemistry Frontiers 8 (2021): 6720–6759.

[25] Z.‐M. Wu , S.‐Z. Liu , X.‐Z. Cheng , W.‐Z. Ding , T. Zhu , and B. Chen , “Recent Progress of On‐resin Cyclization for the Synthesis of Clycopeptidomimetics,” Chinese Chemical Letters 27 (2016): 1731–1739, 10.1016/j.cclet.2016.04.024.

[26] W. Zhan , H. Duan , and C. Li , “Recent Advances in Metal‐Free Peptide Stapling Strategies,” Chem & Bio Engineering 1 (2024): 593–605, 10.1021/cbe.3c00123. PMC11835171 39974699

[27] C. Bechtler and C. Lamers , “Macrocyclization Strategies for Cyclic Peptides and Peptidomimetics,” RSC Medicinal Chemistry 12 (2021): 1325–1351, 10.1039/D1MD00083G. 34447937 PMC8372203

[28] L. Reguera and D. G. Rivera , “Multicomponent Reaction Toolbox for Peptide Macrocyclization and Stapling,” Chemical Reviews 119 (2019): 9836–9860, 10.1021/acs.chemrev.8b00744. 30990310

[29] C. J. White and A. K. Yudin , “Contemporary Strategies for Peptide Macrocyclization,” Nature Chemistry 3 (2011): 509–524, 10.1038/nchem.1062. 21697871

[30] P. Fang , W.‐K. Pang , S. Xuan , W.‐L. Chan , and K. C.‐F. Leung , “Recent Advances in Peptide Macrocyclization Strategies,” Chemical Society Reviews 53 (2024): 11725–11771, 10.1039/D3CS01066J. 39560122

[31] L. R. Malins , “Peptide Modification and Cyclization via Transition‐metal Catalysis,” Current Opinion in Chemical Biology 46 (2018): 25–32, 10.1016/j.cbpa.2018.03.019. 29656180

[32] D. Sun , “Recent Advances in Macrocyclic Drugs and Microwave‐Assisted and/or Solid‐Supported Synthesis of Macrocycles,” Molecules 27 (2022): 1012, 10.3390/molecules27031012. 35164274 PMC8839925

[33] J. He , P. Ghosh , and C. Nitsche , “Biocompatible Strategies for Peptide Macrocyclisation,” Chemical Science 15 (2024): 2300–2322, 10.1039/D3SC05738K. 38362412 PMC10866349

[34] A. M. Webster and S. L. Cobb , “Recent Advances in the Synthesis of Peptoid Macrocycles,” Chemistry – A European Journal 24 (2018): 7560–7573, 10.1002/chem.201705340. 29356125 PMC6001806

[35] V. Sarojini , A. J. Cameron , K. G. Varnava , W. A. Denny , and G. Sanjayan , “Cyclic Tetrapeptides From Nature and Design: A Review of Synthetic Methodologies, Structure, and Function,” Chemical Reviews 119 (2019): 10318–10359, 10.1021/acs.chemrev.8b00737. 31418274

[36] J. Brinkhofer , M. Werner , A. Kokollari , et al., “Late‐Stage Amination of Peptides on the Solid Phase,” Chemistry – A European Journal 31 (2025): e202501229, 10.1002/chem.202501229. 40322874 PMC12172594

[37] S. Pal , J. Openy , A. Krzyzanowski , A. Noisier , and P. ′t Hart , “On‐Resin Photochemical Decarboxylative Arylation of Peptides,” Organic Letters 26 (2024): 2795–2799, 10.1021/acs.orglett.3c03070. 37819674 PMC11019635

[38] M. Werner , J. Brinkhofer , L. Hammermüller , et al., “Peptide Boronic Acids by Late‐Stage Hydroboration on the Solid Phase,” Advancement of Science 11 (2024): 2400640, 10.1002/advs.202400640. PMC11267286 38810019

[39] N. Jung , M. Wiehn , and S. Bräse , Combinatorial Chemistry on Solid Supports, ed. S. Bräse , (Springer Berlin Heidelberg, 2007), 1–88.

[40] J. Alsina and F. Albericio , “Solid‐phase Synthesis of C‐terminal Modified Peptides,” Peptide Science 71 (2003): 454–477, 10.1002/bip.10492. 14517898

[41] M. Soural , J. Hlaváč , and V. Krchňák , Amino Acids, Peptides and Proteins in Organic Chemistry (Wiley‐VCH, 2010), 273–312.

[42] S. Noki , B. G. de la Torre , and F. Albericio , “Safety‐Catch Linkers for Solid‐Phase Peptide Synthesis,” Molecules 29 (2024): 1429, 10.3390/molecules29071429. 38611709 PMC11012524

[43] P. Grieco , P. M. Gitu , and V. J. Hruby , “Preparation of ‘side‐chain‐to‐side‐chain’ Cyclic Peptides by Allyl and Alloc Strategy: Potential for Library Synthesis,” Journal of Peptide Research 57 (2001): 250–256, 10.1111/j.1399-3011.2001.00816.x. 11298927

[44] K. R. Wilson , S. Sedberry , R. Pescatore , et al., “Microwave‐assisted Cleavage of Alloc and Allyl Ester Protecting Groups in Solid Phase Peptide Synthesis,” Journal of Peptide Science 22 (2016): 622–627, 10.1002/psc.2910. 27501347

[45] P. Napier , N. Bakas , A. Bhat , and A. Noncovich , “Open‐Flask Protocol for the Removal of Alloc Carbamate and Allyl Ester Protecting Groups. Application to In‐solution and On‐resin Peptide Synthesis,” Journal of Organic Chemistry 90 (2025): 197–201, 10.1021/acs.joc.4c02115. 39668698

[46] M. Werner , J. Pampel , T. L. Pham , and F. Thomas , “Late‐Stage Functionalisation of Peptides on the Solid Phase by an Iodination‐Substitution Approach,” Chemistry – A European Journal 28 (2022): e202201339, 10.1002/chem.202201339. 35700354 PMC9545490

[47] R. J. Spears , C. McMahon , and V. Chudasama , “Cysteine Protecting Groups: Applications in Peptide and Protein Science,” Chemical Society Reviews 50 (2021): 11098–11155, 10.1039/D1CS00271F. 34605832

[48] A. Chakraborty , S. N. Mthembu , B. G. de la Torre , and F. Albericio , “Ready to Use Cysteine Thiol Protecting Groups in SPPS, A Practical Overview,” Organic Process Research & Development 28 (2024): 26–45, 10.1021/acs.oprd.3c00425.

[49] X. Sun , F. Ye , D. Hu , and P. Wang , “Sustainable Peptide Synthesis by Photoredox‐Catalyzed Picoc‐SPPS,” Journal of the American Chemical Society 147 (2025): 48244–48253, 10.1021/jacs.5c17715. 41392940

[50] P. Klán , T. Šolomek , C. G. Bochet , et al., “Photoremovable Protecting Groups in Chemistry and Biology: Reaction Mechanisms and Efficacy,” Chemical Reviews 113 (2013): 119–191, 10.1021/cr300177k. 23256727 PMC3557858

[51] S. R. Chhabra , B. Hothi , D. J. Evans , P. D. White , B. W. Bycroft , and W. C. Chan , “An Appraisal of New Variants of Dde Amine Protecting Group for Solid Phase Peptide Synthesis,” Tetrahedron Letters 39 (1998): 1603–1606, 10.1016/S0040-4039(97)10828-0.

[52] W. Tang and M. L. Becker , ““Click” Reactions: A Versatile Toolbox for the Synthesis of Peptide‐conjugates,” Chemical Society Reviews 43 (2014): 7013–7039, 10.1039/C4CS00139G. 24993161

[53] J. M. J. M. Ravasco , H. Faustino , A. Trindade , and P. M. P. Gois , “Bioconjugation With Maleimides: A Useful Tool for Chemical Biology,” Chemistry – A European Journal 25 (2019): 43–59. 30095185 10.1002/chem.201803174

[54] K. Renault , J. W. Fredy , P.‐Y. Renard , and C. Sabot , “Covalent Modification of Biomolecules Through Maleimide‐Based Labeling Strategies,” Bioconjugate Chemistry 29 (2018): 2497–2513, 10.1021/acs.bioconjchem.8b00252. 29954169

[55] S. B. Gunnoo and A. Madder , “Chemical Protein Modification Through Cysteine,” Chembiochem 17 (2016): 529–553, 10.1002/cbic.201500667. 26789551

[56] E. Calce , M. Leone , F. A. Mercurio , L. Monfregola , and S. De Luca , “Solid‐Phase S‐Alkylation Promoted by Molecular Sieves,” Organic Letters 17 (2015): 5646–5649, 10.1021/acs.orglett.5b02931. 26523342

[57] D. R. Shinde , S. M. Bodake , and U. K. Marelli , “Peptide Functionalization With Dithioate and Trithioate Groups: A CS2‐Mediated Solid‐Phase Approach,” Organic Letters 27 (2025): 6271–6278, 10.1021/acs.orglett.5c01282. 40322964

[58] J. Lee , J. H. Griffin , and T. I. Nicas , “Solid‐Phase Total Synthesis of Bacitracin A,” The Journal of Organic Chemistry 61 (1996): 3983–3986, 10.1021/jo960580b. 11667271

[59] Y. Liu , G. Li , W. Ma , et al., “Late‐stage Peptide Modification and Macrocyclization Enabled by Tertiary Amine Catalyzed Tryptophan Allylation,” Chemical Science 15 (2024): 11099–11107, 10.1039/D4SC01244E. 39027288 PMC11253200

[60] S. Dutta , A. Chowdhury , and A. Bandyopadhyay , “Introducing Chemoselective Peptide Conjugation via N‐Alkylation of Pyridyl‐alanine: Solution and Solid Phase Applications,” Organic Letters 26 (2024): 8206–8210, 10.1021/acs.orglett.4c03168. 39269272

[61] T. R. White , C. M. Renzelman , A. C. Rand , et al., “On‐resin N‐methylation of Cyclic Peptides for Discovery of Orally Bioavailable Scaffolds,” Nature Chemical Biology 7 (2011): 810–817, 10.1038/nchembio.664. 21946276 PMC3210067

[62] J. Bertran‐Vicente , M. Schümann , P. Schmieder , E. Krause , and C. P. R. Hackenberger , “Direct Access to Site‐specifically Phosphorylated‐lysine Peptides From a Solid‐support,” Organic & Biomolecular Chemistry 13 (2015): 6839–6843, 10.1039/C5OB00734H. 26018866

[63] X. Liu , L. R. Malins , M. Roche , et al., “Site‐Selective Solid‐Phase Synthesis of a CCR5 Sulfopeptide Library To Interrogate HIV Binding and Entry,” ACS Chemical Biology 9 (2014): 2074–2081. 24963694 10.1021/cb500337r PMC4168781

[64] Z. He , Y. Liu , G. Bao , et al., “Intermolecular Sulfur Atom Transfer Cascade Enabled Late‐stage Introduction of Sulfilimines Into Peptides,” Chemical Science 15 (2024): 17058–17063, 10.1039/D4SC02166E. 39345762 PMC11425069

[65] A. J. Cameron , P. W. R. Harris , and M. A. Brimble , “ *On‐*Resin Preparation of Allenamidyl Peptides: A Versatile Chemoselective Conjugation and Intramolecular Cyclisation Tool,” Angewandte Chemie International Edition 59 (2020): 18054–18061, 10.1002/anie.202004656. 32700356

[66] S. Ficht , R. J. Payne , R. T. Guy , and C.‐H. Wong , “Solid‐Phase Synthesis of Peptide and Glycopeptide Thioesters Through Side‐Chain‐Anchoring Strategies,” Chemistry – A European Journal 14 (2008): 3620–3629, 10.1002/chem.200701978. 18278777

[67] H. E. Elashal , R. D. Cohen , and M. Raj , “Fmoc Solid‐phase Synthesis of C‐terminal Modified Peptides by Formation of a Backbone Cyclic Urethane Moiety,” Chemical Communications 52 (2016): 9699–9702, 10.1039/C6CC04245G. 27407005

[68] A. Selvaraj , H.‐T. Chen , A. Ya‐Ting Huang , and C.‐L. Kao , “Expedient On‐resin Modification of a Peptide C‐terminus Through a Benzotriazole Linker,” Chemical Science 9 (2018): 345–349, 10.1039/C7SC03229C. 29629103 PMC5868309

[69] M. Shang , J. He , M. G. Gardiner , and C. Nitsche , “Biocompatible Synthesis of Macrocyclic Thiazole Peptides From Chiral α‐amino Nitriles,” Organic & Biomolecular Chemistry 23 (2025): 9815–9818, 10.1039/D4OB01989J. 39775491

[70] L. Quinti , R. Weissleder , and C.‐H. Tung , “A Fluorescent Nanosensor for Apoptotic Cells,” Nano Letters 6 (2006): 488–490, 10.1021/nl0524694. 16522048

[71] D. Chakraborty , K. Islam , and M. Luo , “Facile Synthesis and Altered Ionization Efficiency of Diverse Nε‐alkyllysine‐containing Peptides,” Chemical Communications 48 (2012): 1514–1516, 10.1039/C1CC14711K. 21959946 PMC3573693

[72] K. Pels and T. Kodadek , “Solid‐Phase Synthesis of Diverse Peptide Tertiary Amides by Reductive Amination,” ACS Combinatorial Science 17 (2015): 152–155, 10.1021/acscombsci.5b00007. 25695359 PMC4447181

[73] J. Morimoto , Y. Fukuda , and S. Sando , “Solid‐Phase Synthesis of β‐Peptoids With Chiral Backbone Substituents Using Reductive Amination,” Organic Letters 19 (2017): 5912–5915, 10.1021/acs.orglett.7b02909. 29039680

[74] V. E. Zwicker , B. L. Oliveira , J. H. Yeo , et al., “A Fluorogenic Probe for Cell Surface Phosphatidylserine Using an Intramolecular Indicator Displacement Sensing Mechanism,” Angewandte Chemie, International Edition 58 (2019): 3087–3091, 10.1002/anie.201812489. 30548909

[75] Y. Zhou , Y. Chen , and C. He , “Solid‐phase Synthesis of Peptide Mn( i )–carbonyl Bioconjugates and Their CO Release Upon Visible Light Activation,” Dalton Transactions 50 (2021): 4231–4236, 10.1039/D1DT00395J. 33687425

[76] J. Luo and C. He , “Chemical Protein Synthesis Enabled Engineering of Saccharide Oxidative Cleavage Activity in Artificial Metalloenzymes,” International Journal of Biological Macromolecules 256 (2024): 128083, 10.1016/j.ijbiomac.2023.128083. 38000595

[77] H. Wu , G. Mousseau , S. Mediouni , S. T. Valente , and T. Kodadek , “Cell‐Permeable Peptides Containing Cycloalanine Residues,” Angewandte Chemie International Edition 55 (2016): 12637–12642, 10.1002/anie.201605745. 27529332 PMC5220248

[78] B. Emenike , S. Shahin , and M. Raj , “Bioinspired Synthesis of Allysine for Late‐Stage Functionalization of Peptides,” Angewandte Chemie, International Edition 63 (2024): e202403215, 10.1002/anie.202403215. 38529755 PMC11254099

[79] L. Reguera , Y. Méndez , A. R. Humpierre , O. Valdés , and D. G. Rivera , “Multicomponent Reactions in Ligation and Bioconjugation Chemistry,” Accounts of Chemical Research 51 (2018): 1475–1486, 10.1021/acs.accounts.8b00126. 29799718

[80] F. E. Morales , H. E. Garay , D. F. Muñoz , et al., “Aminocatalysis‐Mediated on‐Resin Ugi Reactions: Application in the Solid‐Phase Synthesis of N‐Substituted and Tetrazolo Lipopeptides and Peptidosteroids,” Organic Letters 17 (2015): 2728–2731, 10.1021/acs.orglett.5b01147. 25994574

[81] M. G. Ricardo , D. Llanes , L. A. Wessjohann , and D. G. Rivera , “Introducing the Petasis Reaction for Late‐Stage Multicomponent Diversification, Labeling, and Stapling of Peptides,” Angewandte Chemie, International Edition 58 (2019): 2700–2704, 10.1002/anie.201812620. 30589179

[82] S. Krajcovicova and D. R. Spring , “Tryptophan in Multicomponent Petasis Reactions for Peptide Stapling and Late‐Stage Functionalisation,” Angewandte Chemie, International Edition 62 (2023): e202307782. 37389988 10.1002/anie.202307782

[83] B. Li , J. Parker , J. Tong , and T. Kodadek , “Synthesis of Membrane‐Permeable Macrocyclic Peptides via Imidazopyridinium Grafting,” Journal of the American Chemical Society 146 (2024): 14633–14644, 10.1021/jacs.4c01920. 38752889 PMC11705912

[84] S. A. Abboud and T. Kodadek , “2‐Pyridone Formation: An Efficient Method for the Solid‐Phase Synthesis of Homodimers,” Chemistry – A European Journal 30 (2024): e202302937, 10.1002/chem.202302937. 37939246 PMC10843674

[85] S. A. Abboud and T. Kodadek , “Solid‐Phase Synthesis of Diverse Macrocycles by Regiospecific 2‐Pyridone Formation: Scope and Applications,” JACS Au 4 (2024): 3018–3027, 10.1021/jacsau.4c00352. 39211620 PMC11350735

[86] M. Kamalov , P. W. R. Harris , J. M. Wood , and M. A. Brimble , “On Resin Synthesis and Cross‐linking of Collagen Peptides Containing the Advanced Glycation End‐product Pyrraline via Maillard Condensation,” Chemical Communications 51 (2015): 9475–9478, 10.1039/C5CC03052H. 25963401

[87] E. Masri , Ahsanullah , M. Accorsi , and J. Rademann , “Side‐Chain Modification of Peptides Using a Phosphoranylidene Amino Acid,” Organic Letters 22 (2020): 2976–2980, 10.1021/acs.orglett.0c00713. 32223201

[88] J. W. Meringdal and D. Menche , “Suzuki–Miyaura (hetero‐)aryl Cross‐coupling: Recent Findings and Recommendations,” Chemical Society Reviews 54 (2025): 5746–5765, 10.1039/D4CS01108B. 40392002

[89] A. J. J. Lennox and G. C. Lloyd‐Jones , “Selection of Boron Reagents for Suzuki–Miyaura Coupling,” Chemical Society Reviews 43 (2014): 412–443, 10.1039/C3CS60197H. 24091429

[90] T. Willemse , W. Schepens , H. W. T. V. Vlijmen , B. U. W. Maes , and S. Ballet , “The Suzuki–Miyaura Cross‐Coupling as a Versatile Tool for Peptide Diversification and Cyclization,” Catalysts 7 (2017): 74, 10.3390/catal7030074.

[91] N.‐D. Doan , S. Bourgault , M. Létourneau , and A. Fournier , “Effectiveness of the Suzuki−Miyaura Cross‐Coupling Reaction for Solid‐Phase Peptide Modification,” Journal of Combinatorial Chemistry 10 (2008): 44–51, 10.1021/cc700128b. 18067269

[92] B. E. Haug , W. Stensen , and J. S. Svendsen , “Application of the Suzuki–Miyaura Cross‐coupling to Increase Antimicrobial Potency Generates Promising Novel Antibacterials,” Bioorganic & Medicinal Chemistry Letters 17 (2007): 2361–2364, 10.1016/j.bmcl.2006.12.049. 17350833

[93] M. Limbach , M. Löweneck , J. V. Schreiber , J. Frackenpohl , D. Seebach , and A. Billich , “Synthesis of β3‐Homophenylalanine‐Derived Amino Acids and Peptides by Suzuki Coupling in Solution and on Solid Support,” Helvetica Chimica Acta 89 (2006): 1427–1441, 10.1002/hlca.200690143.

[94] A. Afonso , O. Cussó , L. Feliu , and M. Planas , “Solid‐Phase Synthesis of Biaryl Cyclic Peptides Containing a 3‐Aryltyrosine,” European Journal of Organic Chemistry 2012 (2012): 6204–6211, 10.1002/ejoc.201200832. PMC6444451 30992724

[95] A. Afonso , C. Rosés , M. Planas , and L. Feliu , “Biaryl Peptides From 4‐Iodophenylalanine by Solid‐Phase Borylation and Suzuki–Miyaura Cross‐Coupling,” European Journal of Organic Chemistry 2010 (2010): 1461–1468, 10.1002/ejoc.200901350.

[96] S. Li , D. Pissarnitski , T. Nowak , M. Wleklinski , and S. W. Krska , “Merging Late‐Stage Diversification With Solid‐Phase Peptide Synthesis Enabled by High‐Throughput on‐Resin Reaction Screening,” ACS Catalysis 12 (2022): 3201–3210, 10.1021/acscatal.1c05502.

[97] O. A. Shepperson , M. A. Malone , K. I. M. Arnott , R. J. Brown , and A. G. Jamieson , “On‐Resin Synthesis and Late‐Stage Functionalization of Macrocyclic Atosiban Mimetics via 5‐Iodo‐1,4‐triazoles,” Organic Letters 27 (2025): 11249–11253. 41003678 10.1021/acs.orglett.5c03507 PMC12519475

[98] R. Chinchilla and C. Nájera , “The Sonogashira Reaction: A Booming Methodology in Synthetic Organic Chemistry,” Chemical Reviews 107 (2007): 874–922, 10.1021/cr050992x. 17305399

[99] F. Yan , X. Zhang , D. Li , N. Zhu , and H. Bao , “Recent Applications of the Sonogashira Reaction in the Synthesis of Drugs and Their Derivatives: A Review,” Applied Organometallic Chemistry 39 (2025): e7932, 10.1002/aoc.7932.

[100] N.‐D. Doan , M. Poujol de Molliens , M. Létourneau , A. Fournier , and D. Chatenet , “Optimization of On‐resin Palladium‐catalyzed Sonogashira Cross‐coupling Reaction for Peptides and its Use in a Structure–activity Relationship Study of a Class B GPCR Ligand,” European Journal of Medicinal Chemistry 104 (2015): 106–114, 10.1016/j.ejmech.2015.09.017. 26448038

[101] C. Doebelin , I. Bertin , S. Schneider , et al., “Development of Dipeptidic hGPR54 Agonists,” ChemMedChem 11 (2016): 2147–2154. 27562608 10.1002/cmdc.201600331

[102] P. Yang , M. J. Širvinskas , B. Li , et al., “Teraryl Braces in Macrocycles: Synthesis and Conformational Landscape Remodeling of Peptides,” Journal of the American Chemical Society 145 (2023): 13968–13978, 10.1021/jacs.3c03512. 37326500

[103] J. C. Twitty , Y. Hong , B. Garcia , et al., “Diversifying Amino Acids and Peptides via Deaminative Reductive Cross‐Couplings Leveraging High‐Throughput Experimentation,” Journal of the American Chemical Society 145 (2023): 5684–5695, 10.1021/jacs.2c11451. 36853652 PMC10117303

[104] J. N. deGruyter , L. R. Malins , L. Wimmer , et al., “CITU: A Peptide and Decarboxylative Coupling Reagent,” Organic Letters 19 (2017): 6196–6199, 10.1021/acs.orglett.7b03121. 29115835 PMC5792187

[105] T. Qin , J. Cornella , C. Li , et al., “A General Alkyl‐alkyl Cross‐coupling Enabled by Redox‐active Esters and Alkylzinc Reagents,” Science 352 (2016): 801–805, 10.1126/science.aaf6123. 27103669 PMC4867118

[106] A. S. Barahdia , K. L. Thakare , L. Kaur , and R. Jain , “Endogenous Group‐Directed Late‐Stage C−H Functionalization of Peptides,” Advanced Synthesis & Catalysis 366 (2024): 2844–2858, 10.1002/adsc.202400373.

[107] T. Brandhofer and O. García Mancheño , “Site‐Selective C–H Bond Activation/Functionalization of Alpha‐Amino Acids and Peptide‐Like Derivatives,” European Journal of Organic Chemistry 2018 (2018): 6050–6067, 10.1002/ejoc.201800896.

[108] J. Liu , P. Wang , Z. Yan , J. Yan , Kenry , and Q. Zhu , “Recent Advances in Late‐Stage Construction of Stapled Peptides via C−H Activation,” Chembiochem 22 (2021): 2762–2771, 10.1002/cbic.202100044. 33949069

[109] A. F. M. Noisier and M. A. Brimble , “C–H Functionalization in the Synthesis of Amino Acids and Peptides,” Chemical Reviews 114 (2014): 8775–8806, 10.1021/cr500200x. 25144592

[110] W. Wang , M. M. Lorion , J. Shah , A. R. Kapdi , and L. Ackermann , “Late‐Stage Peptide Diversification by Position‐Selective C−H Activation,” Angewandte Chemie, International Edition 57 (2018): 14700–14717, 10.1002/anie.201806250. 29969532

[111] B.‐B. Zhan , M.‐X. Jiang , and B.‐F. Shi , “Late‐stage Functionalization of Peptides via a Palladium‐catalyzed C(sp 3 )–H Activation Strategy,” Chemical Communications 56 (2020): 13950–13958, 10.1039/D0CC06133F. 33118547

[112] A. Schischko , N. Kaplaneris , T. Rogge , G. Sirvinskaite , J. Son , and L. Ackermann , “Late‐stage Peptide C–H Alkylation for Bioorthogonal C–H Activation Featuring Solid Phase Peptide Synthesis,” Nature Communications 10 (2019): 3553, 10.1038/s41467-019-11395-3. PMC6685959 31391461

[113] X.‐X. Ding , B.‐T. Li , and L. Dong , “Late‐Stage C–H Functionalization of Dehydroalanine‐Containing Peptides With Arylthianthrenium Salts and Its Application in Synthesis of Tentoxin Analogue,” Organic Letters 27 (2025): 863–868, 10.1021/acs.orglett.4c04535. 39808515

[114] V. Castro , H. Rodríguez , and F. Albericio , “CuAAC: An Efficient Click Chemistry Reaction on Solid Phase,” ACS Combinatorial Science 18 (2016): 1–14, 10.1021/acscombsci.5b00087. 26652044

[115] S. M. Bodake and U. K. Marelli , “Ketenimine Multicomponent Strategy for Multifaceted Amidine Functionalization of Peptides on the Solid Phase,” Angewandte Chemie, International Edition 64 (2025): e202509854, 10.1002/anie.202509854. 40734244

[116] Z. He , X. Zhao , W.‐Y. Gao , et al., “Controlled Reversible Methionine‐selective Sulfimidation of Peptides,” Science Advances 11 (2025): eadv8712, 10.1126/sciadv.adv8712. 40397727 PMC12094199

[117] Y. Liu , Z. He , W. Ma , et al., “Copper(I)‐Catalyzed Late‐Stage Introduction of Oxime Ethers Into Peptides at the Carboxylic Acid Site,” Organic Letters 24 (2022): 9248–9253, 10.1021/acs.orglett.2c03813. 36508502

[118] J. Plescia and N. Moitessier , “Design and Discovery of Boronic Acid Drugs,” European Journal of Medicinal Chemistry 195 (2020): 112270, 10.1016/j.ejmech.2020.112270. 32302879

[119] I. Jesin and G. C. Nandi , “Recent Advances in the A3 Coupling Reactions and Their Applications,” European Journal of Organic Chemistry 2019 (2019): 2704–2720, 10.1002/ejoc.201900001.

[120] K. Van holsbeeck , M. Elsocht , and S. Ballet , “Propargylamine Amino Acids as Constrained Nε‐Substituted Lysine Mimetics,” Organic Letters 25 (2023): 130–133. 36546856 10.1021/acs.orglett.2c03931

[121] J. Zhang , C. Proulx , A. Tomberg , and W. D. Lubell , “Multicomponent Diversity‐Oriented Synthesis of Aza‐Lysine‐Peptide Mimics,” Organic Letters 16 (2014): 298–301, 10.1021/ol403297v. 24328523

[122] J. Zhang , M. Mulumba , H. Ong , and W. D. Lubell , “Diversity‐Oriented Synthesis of Cyclic Azapeptides by A 3 ‐Macrocyclization Provides High‐Affinity CD36‐Modulating Peptidomimetics,” Angewandte Chemie, International Edition 56 (2017): 6284–6288, 10.1002/anie.201611685. 28090719

[123] T. Qin , L. R. Malins , J. T. Edwards , et al., “Nickel‐Catalyzed Barton Decarboxylation and Giese Reactions: A Practical Take on Classic Transforms,” Angewandte Chemie, International Edition 56 (2017): 260–265, 10.1002/anie.201609662. 27981703 PMC5295468

[124] Y. E. Jad , S. K. Gudimella , T. Govender , B. G. de la Torre , and F. Albericio , “Solid‐Phase Synthesis of Pyrrole Derivatives Through a Multicomponent Reaction Involving Lys‐Containing Peptides,” ACS Combinatorial Science 20 (2018): 187–191, 10.1021/acscombsci.8b00006. 29444402

[125] J. Petersen , K. E. Christensen , M. T. Nielsen , et al., “Oxidative Modification of Tryptophan‐Containing Peptides,” ACS Combinatorial Science 20 (2018): 344–349, 10.1021/acscombsci.8b00014. 29719155

[126] X.‐Y. Liu , X. Ji , C. Heinis , and J. Waser , “Peptide‐Hypervalent Iodine Reagent Chimeras: Enabling Peptide Functionalization and Macrocyclization**,” Angewandte Chemie, International Edition 62 (2023): e202306036, 10.1002/anie.202306036. 37311172

[127] A.‐C. Knall and C. Slugovc , “Inverse Electron Demand Diels–Alder (iEDDA)‐initiated Conjugation: A (high) Potential Click Chemistry Scheme,” Chemical Society Reviews 42 (2013): 5131, 10.1039/c3cs60049a. 23563107

[128] M. Pagel , R. Meier , K. Braun , M. Wiessler , and A. G. Beck‐Sickinger , “On‐resin Diels–Alder Reaction With Inverse Electron Demand: An Efficient Ligation Method for Complex Peptides With a Varying Spacer to Optimize Cell Adhesion,” Organic & Biomolecular Chemistry 14 (2016): 4809–4816, 10.1039/C6OB00314A. 27117044

[129] Q. Zuo , X. Song , J. Yan , et al., “Triazination/IEDDA Cascade Modular Strategy Installing Pyridines/Pyrimidines Onto Tyrosine Enables Peptide Screening and Optimization,” Journal of the American Chemical Society 147 (2025): 9576–9589, 10.1021/jacs.4c17615. 39885681

[130] S. S. M. Kamble , S. M. Bodake , and U. K. Marelli , “Peptide‐Triazolobenzodiazepine Hybrids: A Catalyst‐Free on‐Resin Strategy to Build Complex Therapeutic Motifs Into Peptides,” Chemistry – A European Journal 31 (2025): e202500836, 10.1002/chem.202500836. 40237132

[131] G. Bao , P. Wang , G. Li , et al., “1,3‐Dipolar Cycloaddition Between Dehydroalanines and C,N‐Cyclic Azomethine Imines: Application to Late‐Stage Peptide Modification,” Angewandte Chemie, International Edition 60 (2021): 5331–5338, 10.1002/anie.202012523. 33179384

[132] I. S. De Jesus , J. A. C. Vélez , E. F. Pissinati , J. T. M. Correia , D. G. Rivera , and M. W. Paixao , “Recent Advances in Photoinduced Modification of Amino Acids, Peptides, and Proteins,” The Chemical Record 24 (2024): e202300322. 38279622 10.1002/tcr.202300322

[133] M. Elkhalifa , M. B. Elbaum , D. M. Chenoweth , and G. A. Molander , “Solid‐Phase Photochemical Decarboxylative Hydroalkylation of Peptides,” Organic Letters 23 (2021): 8219–8223, 10.1021/acs.orglett.1c02928. 34648297 PMC8919077

[134] M. B. Elbaum , M. A. Elkhalifa , G. A. Molander , and D. M. Chenoweth , “Solid‐Phase Photochemical Peptide Homologation Cyclization,” Organic Letters 24 (2022): 5176–5180, 10.1021/acs.orglett.2c02012. 35816696 PMC10435287

[135] J. Openy , G. Amrahova , J.‐Y. Chang , A. Noisier , and P. T. Hart , “Solid‐Phase Peptide Modification via Deaminative Photochemical Csp 3 ‐Csp 3 Bond Formation Using Katritzky Salts,” Chemistry – A European Journal 28 (2022): e202201121, 10.1002/chem.202201121. 35438838 PMC9401037

[136] G. Bao , P. Wang , X. Guo , et al., “Visible‐Light Mediated Deoxygenation of Carboxylic Acid for Late‐Stage Peptide Modification Targeting Dehydroalanine,” Organic Letters 25 (2023): 8338–8343. 37966281 10.1021/acs.orglett.3c03453

[137] J. A. C. Delgado , Y.‐M. Tian , M. Marcon , B. König , and M. W. Paixão , “Side‐Selective Solid‐Phase Metallaphotoredox N(in)‐Arylation of Peptides,” Journal of the American Chemical Society 145 (2023): 26452–26462, 10.1021/jacs.3c10792. 37976043

[138] H. Liu , G. Li , Z. Peng , et al., “Tagging Peptides With a Redox Responsive Fluorescent Probe Enabled by Photoredox Difunctionalization of Phenylacetylenes With Sulfinates and Disulfides,” JACS Au 2 (2022): 2821–2829, 10.1021/jacsau.2c00577. 36590269 PMC9795567

[139] P. R. Choudhury , S. K. Mishra , S. Yadav , S. Singh , and P. Mathur , “Silico Peptide Design: Methods, Resources, and Role of AI,” Journal of Peptide Science 31 (2025): e70063. 41168660 10.1002/psc.70063

[140] D. Hudson , “Matrix Assisted Synthetic Transformations: A Mosaic of Diverse Contributions. I. The Pattern Emerges,” Journal of Combinatorial Chemistry 1 (1999): 333–360, 10.1021/cc990022l. 10748732

[141] J. Lu and P. H. Toy , “Organic Polymer Supports for Synthesis and for Reagent and Catalyst Immobilization,” Chemical Reviews 109 (2009): 815–838, 10.1021/cr8004444. 19128147

[142] C. Blackburn , “Polymer Supports for Solid‐phase Organic Synthesis,” Peptide Science 47 (1998): 311–351, 10.1002/(SICI)1097-0282(1998)47:5<311::AID-BIP2>3.0.CO;2-V.

[143] F. Garcia and F. Albericio , “Solid Supports for the Synthesis of Peptides,” Chimica Oggi 26 (2008): 29.

[144] M. Economidou , N. Mistry , K. M. P. Wheelhouse , and D. M. Lindsay , “Palladium Extraction Following Metal‐Catalyzed Reactions: Recent Advances and Applications in the Pharmaceutical Industry,” Organic Process Research & Development 27 (2023): 1585–1615, 10.1021/acs.oprd.3c00210.

[145] W. P. Gallagher and A. Vo , “Dithiocarbamates: Reagents for the Removal of Transition Metals From Organic Reaction Media,” Organic Process Research & Development 19 (2015): 1369–1373, 10.1021/op500336h.

[146] S. B. H. Kent , “Fundamental Aspects of SPPS and Green Chemical Peptide Synthesis,” Journal of Peptide Science 31 (2025): e70013, 10.1002/psc.70013. 40210223 PMC11985259

[147] O. Al Musaimi , B. G. de la Torre , and F. Albericio , “Greening Fmoc/tBu Solid‐phase Peptide Synthesis,” Green Chemistry 22 (2020): 996–1018.

[148] V. Martin , P. H. G. Egelund , H. Johansson , S. Thordal Le Quement , F. Wojcik , and D. Sejer Pedersen , “Greening the Synthesis of Peptide Therapeutics: An Industrial Perspective,” RSC Advances 10 (2020): 42457–42492, 10.1039/D0RA07204D. 35516773 PMC9057961

[149] A. Kokollari , M. Werner , C. Lindner , T. L. Pham , and F. Thomas , “Rapid On‐Resin N ‐Formylation of Peptides as One‐Pot Reaction,” Chembiochem 24 (2023): e202300571, 10.1002/cbic.202300571. 37695727

[150] K. G. Varnava and V. Sarojini , “Making Solid‐Phase Peptide Synthesis Greener: A Review of the Literature,” Chemistry ‐ An Asian Journal 14 (2019): 1088–1097, 10.1002/asia.201801807. 30681290

[151] A. El‐Faham and F. Albericio , “Peptide Coupling Reagents, More Than a Letter Soup,” Chemical Reviews 111 (2011): 6557–6602, 10.1021/cr100048w. 21866984

[152] D. S. M. M. Jaradat , O. Al Musaimi , and F. Albericio , “Advances in Solid‐phase Peptide Synthesis in Aqueous Media (ASPPS),” Green Chemistry 24 (2022): 6360–6372.

[153] D. Orain , J. Ellard , and M. Bradley , “Protecting Groups in Solid‐Phase Organic Synthesis,” Journal of Combinatorial Chemistry 4 (2002): 1–16, 10.1021/cc0001093. 11790135

[154] S. V. Moradi , W. M. Hussein , P. Varamini , P. Simerska , and I. Toth , “Glycosylation, an Effective Synthetic Strategy to Improve the Bioavailability of Therapeutic Peptides,” Chemical Science 7 (2016): 2492–2500, 10.1039/C5SC04392A. 28660018 PMC5477030

[155] M. Muttenthaler , F. Albericio , and P. E. Dawson , “Methods, Setup and Safe Handling for Anhydrous Hydrogen Fluoride Cleavage in Boc Solid‐phase Peptide Synthesis,” Nature Protocols 10 (2015): 1067–1083, 10.1038/nprot.2015.061. 26086408

[156] S. Knauer , N. Koch , C. Uth , R. Meusinger , O. Avrutina , and H. Kolmar , “Sustainable Peptide Synthesis Enabled by a Transient Protecting Group,” Angewandte Chemie, International Edition 59 (2020): 12984–12990, 10.1002/anie.202003676. 32324944 PMC7496111

[157] M. Amblard , J.‐A. Fehrentz , J. Martinez , and G. Subra , “Methods and Protocols of Modern Solid Phase Peptide Synthesis,” Molecular Biotechnology 33 (2006): 239–254, 10.1385/MB:33:3:239. 16946453

[158] K. P. Nandhini , M. Alhassan , C. G. L. Veale , F. Albericio , and B. G. de la Torre , “Methionine‐Containing Peptides: Avoiding Secondary Reactions in the Final Global Deprotection,” ACS Omega 8 (2023): 15631–15637, 10.1021/acsomega.3c01058. 37151509 PMC10157837

[159] S. N. Mthembu , A. Chakraborty , R. Schönleber , F. Albericio , and B. G. de la Torre , “TFA Cleavage Strategy for Mitigation of S‐tButylated Cys‐Peptide Formation in Solid‐Phase Peptide Synthesis,” Organic Process Research & Development 29 (2025): 691–703, 10.1021/acs.oprd.4c00443.

[160] J. Pawlas , J. Billing , B. Tebikachew , L. Wahlström , and L. M. Haugaard‐Kedström , “A Sustainable Approach to ε‐Lys Branched GLP‐1 Analogs: Integrating Green SPPS, Metal‐free Alloc Removal, Waste Minimization and TFA/PFAS‐free Resin Cleavage,” Organic Process Research & Development 29 (2025): 2989–2997, 10.1021/acs.oprd.5c00341.

[161] T. Bruckdorfer , O. Marder , and F. Albericio , “From Production of Peptides in Milligram Amounts for Research to Multi‐tons Quantities for Drugs of the Future,” Current Pharmaceutical Biotechnology 5 (2004): 29–43, 10.2174/1389201043489620. 14965208

[162] J. Pawlas and A. Lindgren , “Expanding the Reach of Sustainable Solid‐Phase Peptide Synthesis: One‐Pot, Metal‐Free Alloc Removal–Peptide Coupling,” Organic Letters 27 (2025): 2891–2896, 10.1021/acs.orglett.5c00423. 40094332

