# Supplementary material for: Late‐Stage Functionalization of Peptides on the Solid Phase
Source: Angew Chem Int Ed Engl. 2026 Jun 10;65(31):e4556652. doi: 10.1002/anie.4556652 (PMC13411238; doi:10.1002/anie.4556652)
Supplement: Supplementary file 1 — Supporting File: anie72996‐sup‐0001‐SupMat.pdf. We have added a small guideline of practical considerations as supplementary material. The authors have cited additional references within the Supporting Information [140, 141, 142, 143, 144, 145, 146, 147, 148, 149, 150, 151, 152, 153, 154, 155, 156, 157, 158, 159, 160, 161, 162]. [file ANIE-65-e4556652-s001.pdf]

Table of contents

1 Practical Considerations S2

1.1 Selection of Resins, Linkers and Solvents S2

1.2 Handling of Resin-Bound Peptides S3

1.3 Protecting group strategy S4

2 Experimental Setup of the Baran Lab S8

3 Experimental Setup of the Thomas Lab S9

4 Experimental Setup for On-Resin Photocatalysis S10

5 Screening using well plates S11

6 Literature S12

1 Practical Considerations

1.1 Selection of Resins, Linkers and Solvents

Alongside coupling reagents, protecting group chemistry and solvent choice, the resin and the linker that connects the *C*-terminal amino acid to the resin are the cornerstones of modern peptide synthesis. These topics have been extensively reviewed.[1-7] Therefore this chapter will only provide relevant information on late-stage functionalization.

Peptides are synthesized on an insoluble polymer support.[8-11] These resins can also be considered the solvents of solid-phase synthesis. Figure S1 shows the most common resins, which differ in structure and polarity. This must be taken into consideration when establishing new late-stage modification methods. Resins swell in solvents, allowing reagents to diffuse into their pores. Polystyrene (PS) resin is the most commonly used resin because it is inexpensive; however, it is also nonpolar. It is therefore swellable in nonpolar solvents, but less so in alcohols and water. Conversely, polyethylene (PEG)- and polyacrylamide-based resins are highly versatile, display a higher polarity than polystyrene resin but have a lower loading density. While this is preferable for longer peptides, it can be problematic for scale-up. In general, solvents that lead to high swelling are more suitable for synthesis. For example, if water is intended to be used as the solvent, PS resin is not suitable, and PEG-based resins such as TentaGel are a better choice. Nevertheless, a successful reaction can still be achieved as long as swelling is observed. The amination of iodohomoalanine-containing peptides on PS resin is more effective in acetonitrile than in DMF,[12] even though resin swelling in acetonitrile is lower. Switching from PS to PEG resin may increase swelling, but this does not necessarily increase the yield. PS resin has been shown to give a better yield than PEG-based resins for photocatalytic decarboxylative arylation,[13] but both types of resin can be used for the hydroboration of alkene and alkyne modified peptides.[14]

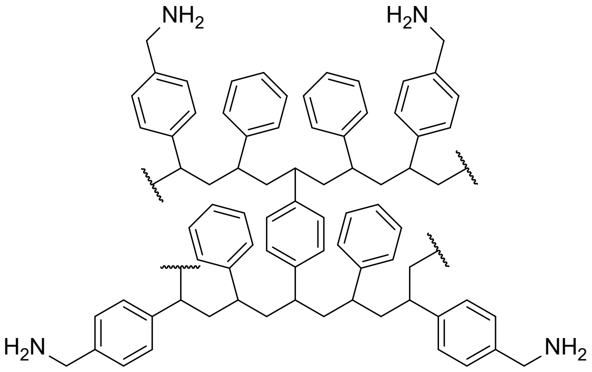

aminomethylated polystyrene crosslinked with divinylbenzene

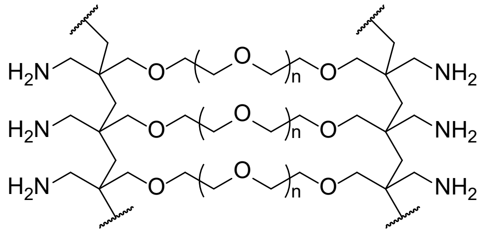

aminomethylated polyethylene glycol

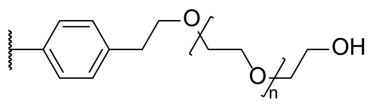

polyethylene glycol grafted polystyrene

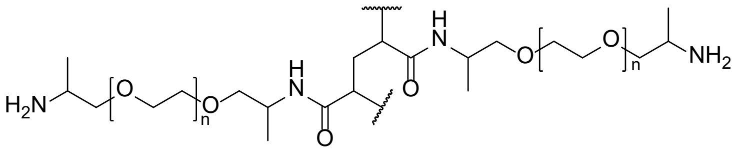

polyethylene glycol grafted polyacrylamide

Figure S1: Common resins in SPPS.

The choice of reagents is also limited by the linker.[15-17] Since the commonly used fluorenylmethoxycarbonyl/*tert*-butyl strategy (Fmoc/*t*-Bu strategy) relies on trifluoroacetic acid (TFA)-sensitive permanent protecting groups, basic conditions can be used but acidic conditions cannot. Trityl-based linkers are highly sensitive to acids, whereas Wang and Rink amide linkers are more stable. If acidic reaction conditions are considered, then linkers suitable for *tert*-butyloxycarbonyl/benzyl strategy (Boc/Bzl strategy) could be used. These tolerate TFA but require HF for cleavage. Alternatively, photocleavable linkers, such as nitrobenzyl, can be used. Finally, safety-catch linkers,[18] which are stable under various conditions, can be considered. Upon activation — including oxidation, reduction or treatment with a distinct reagent — they convert into a cleavable linker. The cleavage conditions affect the types of functionalization that can be introduced. For example, strong acidic conditions may lead to

further modification. Additionally, nucleophilic additives such as triisopropylsilane (TIPS) or thiols can lead to undesired side reactions.

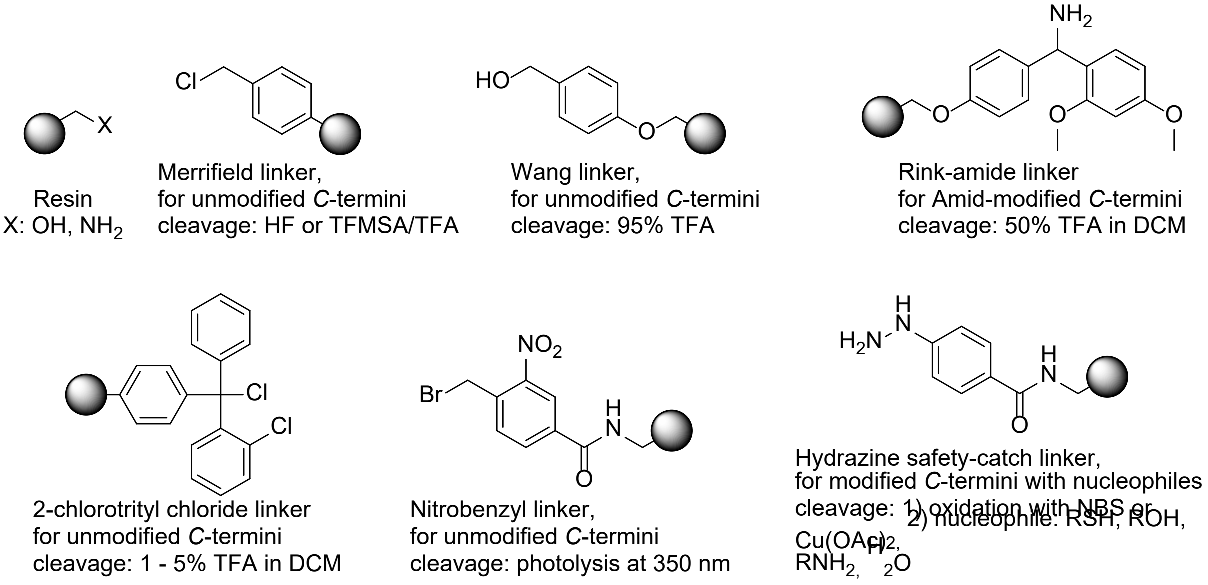

Figure S2: Structures and cleavage conditions of common linkers in SPPS.

1.2 Handling of Resin-Bound Peptides

Late-stage functionalization of fully protected resin-bound peptides has several advantages over the traditional in-solution approach. Fully protected peptides are difficult to dissolve and unprotected peptides are susceptible to side reactions. Once the reaction is finished, the resin can be easily filtered and washed with appropriate solvents to remove the reagents. However, it should be noted that some reagents, particularly transition metal catalysts, can adhere to the resin and cannot be removed by simple washing. In the case of palladium-catalyzed reactions, ammonia solution has been shown to effectively remove the metal catalyst. Solutions of multidentate transition metal chelators can also be used to remove residual metal catalysts from the resin.[13, 19-20]

On-resin late-stage functionalization reactions can in principle be carried out in a borosilicate glass flask with a polytetrafluoroethylene (PTFE) stirring bar. Once the reaction has finished, the resin can be separated by using sintered glass funnels. In our experience, resin beads tend to stick to glass surfaces, including glass pipettes. Labware made of polypropylene (PP), high-density polyethylene (HDPE), low-density polyethylene (LDPE) or polytetrafluoroethylene (PTFE) is a better choice. Small-scale reactions can be performed in PP reaction tubes, which are typically found in biology laboratories. Variants with screw caps are available for a tighter sealing.

Syringe reactors containing a polyethylene (PE) frit are now standard in SPPS and are ideal for late-stage functionalization. Reagents can be dissolved and aspirated into the syringe, which is then capped and placed on a shaker. In case of gas formation, as, for example, observed during on-resin *N*-formylation,[21] open reaction vessels are recommended. Performing reactions in an inert gas atmosphere can be challenging since this is usually associated with Schlenk technique. However, alternative methods have been developed to avoid Schlenk flasks. Ideally, syringe reactors should be handled in a glovebox, but these are not available in every laboratory. The Baran Lab[22] has developed a method, in which the syringe reactor's plunger is removed and the tip is connected to a needle (Figure S3). A flask containing a septum and an argon balloon is then connected. The reagents are added via the syringe under a constant flow of argon. After reconnecting the piston, solvent is added to the flask and aspirated into the syringe. Alternatively, reactions carried out in a syringe reactor or PP reaction tube can be performed in a desiccator containing a nitrogen inlet (Figure 4), which allows the reaction to be performed in a nitrogen or argon atmosphere.

Photocatalysis to functionalize peptides on resin can be performed in glass vials equipped with small PTFE stirrer bars on a magnetic stirrer. Irradiation with a suitable light source can increase the temperature, hence, a fan is required used for cooling (Figure S5).[13]

PP reaction tubes and syringes are ideal for running several reactions in parallel, and these can be agitated on a shaker. Shakers are also available with heating (Figure S4). High-throughput experiments have been performed using 96-well filter plates (Figure S6).[23] First, the resin is added and then the bottom of the plate is sealed. Then, the reagents are added and the top is sealed. After incubation, the seal is removed and filtration is accelerated using a vacuum pump.

1.3 Protecting group strategy

SPPS in general relies on the use of protected amino acid building blocks and therefore this topic has been reviewed extensively.[2, 24] In case of on-resin late-stage functionalization, the peptide is still fully protected, except for the desired reaction site, which is selectively deprotected prior to modification. Therefore, the protecting group of this residue must be orthogonal to the temporary protection group at the α-amino group and the permanent protection groups at the side chains. Initially, Boc has been applied as temporary α-amino protecting group; however, Boc is cleaved under strong acidic conditions and permanent protection required the use of HF sensitive groups such as benzyl-based protecting groups. These conditions are harsh and not compatible with all desired modifications, e.g. glycosidic bonds are not stable under these conditions.[25] Therefore, the Boc/Bzl strategy is only applied under special circumstances, such as the synthesis of highly aggregation prone peptides.[26] The Fmoc/*t*-Bu-strategy proceeds under milder conditions and has become the standard method.[27-28] The α-amino group is temporarily protected with the base-sensitive Fmoc group, which can be removed with secondary amines such as piperidine. Newer developments include the base-sensitive 2,7-disulfo-9-fluorenylmethoxycarbonyl (Smoc) group, which is compatible to SPPS in water[29] and the photocleavable picolyloxycarbonyl (Picoc) group, which is cleaved using a photocatalyst attached to the resin.[30] The functional groups of the side chains are orthogonally protected by acid-sensitive protecting groups, which are removed with concentrated TFA and scavengers during peptide cleavage from the resin. An overview of typical side chain protecting groups is given in Table S1. During acidic cleavage carbocations are formed and can lead to undesired side reactions. This is suppressed by the addition of scavengers such as water, TIPS, ethanedithiol or thioanisole to the final cleavage cocktail.[31-33]

There is a great variety of protecting groups available, which are orthogonal to Fmoc and the acid labile protecting groups and are used to selectively deprotect single amino-acid side chains for on-resin modification. The classic residue for late-stage functionalization is the strong nucleophilic thiol bearing cysteine side chain. Therefore, several cysteine protecting groups have been developed and comprehensively reviewed.[34-35] Other nucleophilic side chains include the amino group bearing lysine and the hydroxy group bearing serine, threonine and tyrosine residues. A very convenient protecting group for these nucleophilic side chains is dimethoxytrityl (Dmt), which can be selectively cleaved within minutes by washing the peptide resin with 1% TFA in dichloromethane.[12, 36] Other highly acid sensitive protecting groups are methyltrityl (Mtt) or monomethoxytrityl (Mmt). Another common alternative class protecting group for nucleophilic side chains are allyl-based protecting groups such as allyloxycarbonyl (Alloc) for amino groups or allyl for carboxylic acids.[37] These are deprotected using $Pd(PPh_3)_4$ and a scavenger such as phenylsilane. The original protocol requires strict exclusion of oxygen, which ideally requires an argon atmosphere.[37] Several improved protocols have been developed to perform the reaction in air.[38-41] Alternatively, the hydrazine sensitive ivDde group (1-(4,4-dimethyl-2,6-dioxocyclohex-1-ylidene)-3-methylbutyl) can be used to protect lysine side chains.[42] Further alternative orthogonal protecting groups are summarized in Table S1. They range from highly acid sensitive protecting groups, over hydrazine, and tetrabutylammonium fluoride (TBAF) sensitive groups to photocleavable groups.

The plethora of orthogonal protecting groups allows on-resin late-stage functionalization at several sites, step-by-step, by choosing an appropriate strategy. Although all Fmoc- and standard side chain protected canonical amino acids are commercially available up to multi-ton scale,[43] this is not the case for other side-chain protecting groups, which require prior synthesis. In terms of chemical elegance and atom economy, a selective reaction on unprotected peptides would be ideal, but this is only possible for a handful of reactions, such as click reactions.[44]

Table S1: Selection of orthogonal protecting groups for SPPS and on-resin late-stage functionalization. If not specified, the protecting group is attached to the amino acid side chain.

Cleavage condition	Protecting group	Amino acids
Base	Fluorenylmethoxycarbonyl<br>(Fmoc)<br>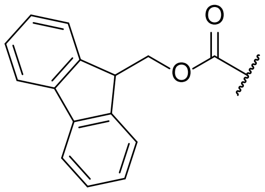	α-amino group
	2,7-Disulfo-9-fluorenylmethoxycarbonyl<br>(Smoc)<br>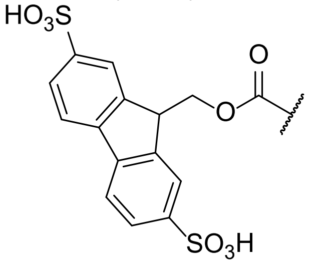	
Concentrated TFA + scavengers	*tert*-Butyl<br>(*t*-Bu)<br>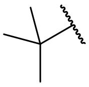	Asp, Glu, Tyr, Ser, Thr
	Trityl<br>(Trt)<br>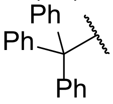	Cys, His, Asn, Gln
	tert-Butyloxycarbonyl<br>(Boc)<br>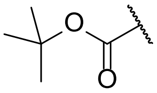	Lys, Trp
	Pentamethyl-2,3-dihydrobenzofuran-5-sulfonyl<br>(Pbf)<br>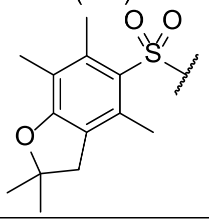	Arg
Diluted TFA	4,4'-Dimethoxytrityl<br>(Dmt)<br>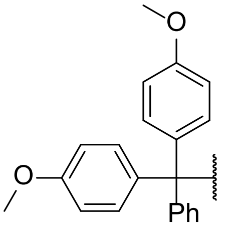	Cys, Ser, Thr, Lys

	2-Phenylisopropyl<br>(2-Ph*i*Pr)<br>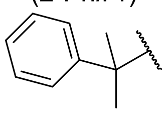	Asp, Glu
Pd(0) + scavengers	Allyloxycarbonyl<br>(Alloc)<br>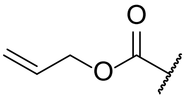<br>Allyl<br>(All)<br>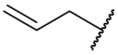	Cys, Lys<br>Asp, Glu, Tyr
Hydrazine	1-(4,4-dimethyl-2,6-dioxocyclohex-1-ylidene)-3-methylbutyl<br>(ivDde)<br>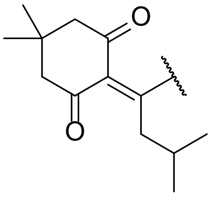<br>4-(*N*-[1-(4,4-dimethyl-2,6-dioxocyclohexylidene)-3-methylbutyl]-amino)benzyl<br>(Dmab)<br>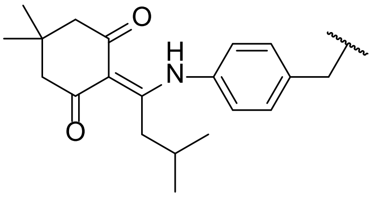	Lys<br>Asp/Glu
Thiols	*tert*-Butylthio<br>(*t*-Bu-S)<br>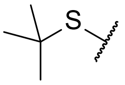<br>Phenyldisulphanylethyloxycarbonyl<br>(Phdec)<br>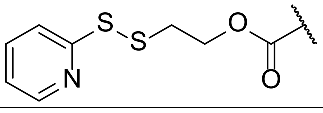	Cys<br>Lys
Photocleavage	Picolyloxycarbonyl<br>(Picoc)<br>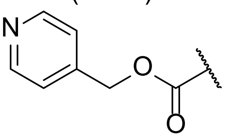<br>*ortho*-Nitrobenzyl<br>(*o*NB)<br>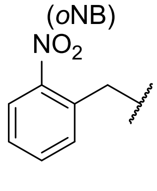	α-amino group<br>Cys

	4,5-Dimethoxy-2-nitrobenzyloxycarbonyl (Dmnbc) 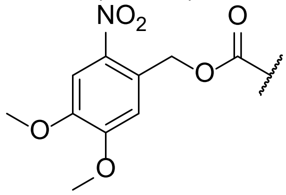 4,5-Dimethoxy-2-nitrobenzyl (Dmnb) 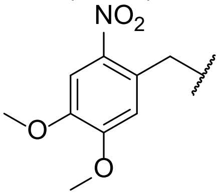	Ser, Thr<br>Asp, Glu
Fluoride (mostly TBAF)	*tert*-Butyldimethylsilyl (TBDMS) 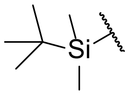 (2-Phenyl-2-trimethylsilyl)ethyl (PTMSE) 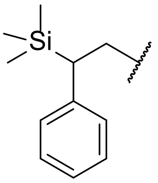	Ser, Thr, Tyr<br>Asp, Glu

2 Experimental Setup of the Baran Lab

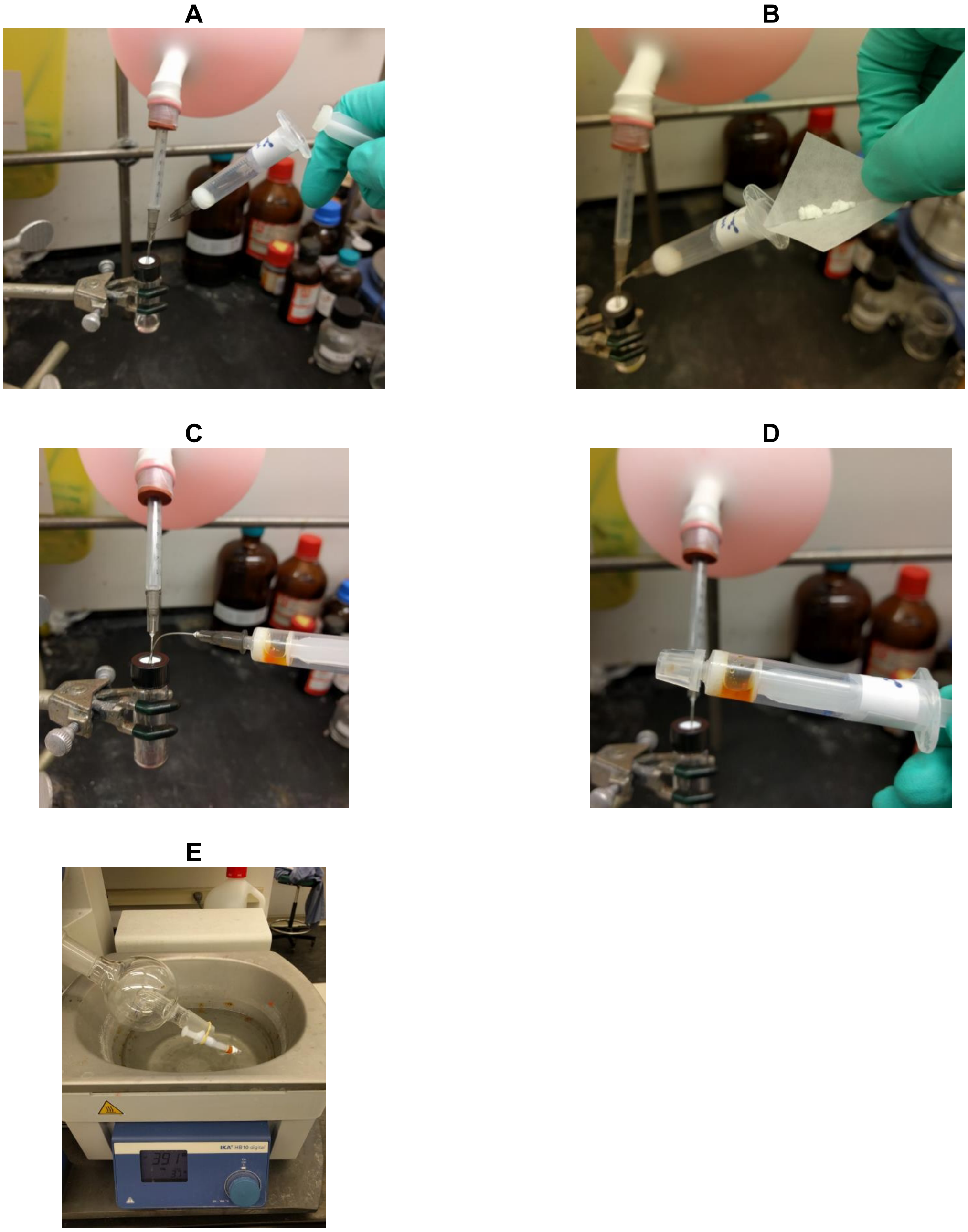

Figure S3: Performing air-sensitive reactions on resin-bound peptides. A) The syringe reactor is connected to a vial equipped with a septum and an argon source (balloon) that contains the reagent solution. At this point, the needle should not come into contact with the reagents. The plunger of the syringe reactor is removed in order to B) add solid reagents in argon counterflow. C) After inserting the plunger, the reagent solutions are drawn from the vial into the syringe. D) The needle is removed and the syringe is sealed. E) A rotary evaporator can be used to agitate and heat the syringe. Sealing the syringe with additional parafilm can prevent penetration of water. Adapted with permission from Quin et al.[22], Copyright © 2017 John Wiley and Sons.

3 Experimental Setup of the Thomas Lab

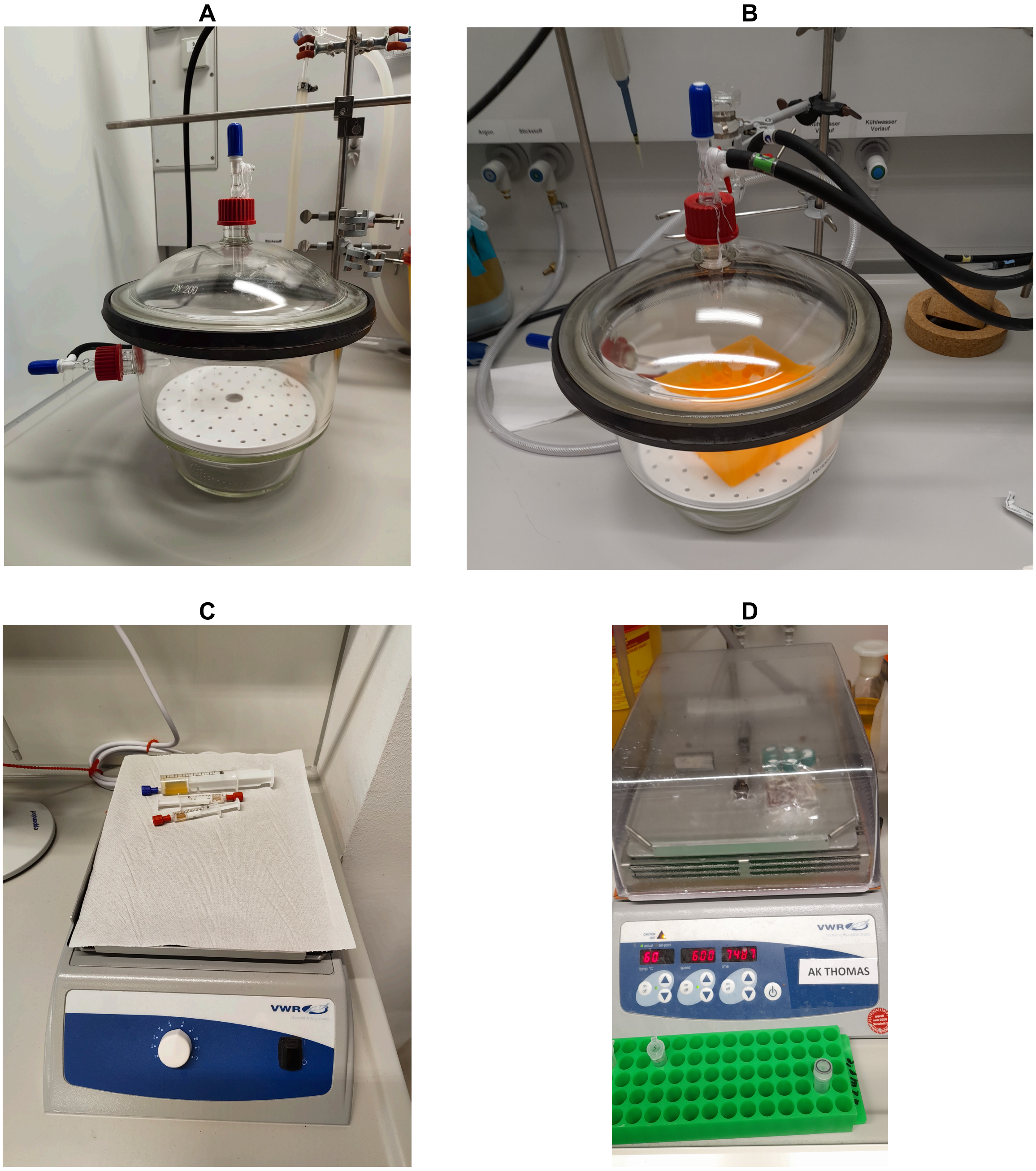

Figure S4: Performing air-sensitive reactions on resin-bound peptides in a parallel format. A) A desiccator connected to a nitrogen line is used for late-stage functionalization on the solid phase in a nitrogen atmosphere. B) In addition, the valve on the lid of the desiccator can be connected to a Schlenk line, which provides vacuum and nitrogen gas from above. With the aid of a rack, reaction samples can be handled in parallel in small glass vials or PP reaction tubes as well as in syringe reactors. Reaction and reagent solutions are handled using syringes or micropipettes. To handle the reaction samples, the lid is removed in a stream of nitrogen through the side valve to ensure a constant nitrogen atmosphere. C) Syringe reactors can be agitated with a shaker, allowing several reactions to be carried out in parallel. D) Shakers with a heating function enable several reactions to be carried out in parallel at elevated temperatures. Please note: Volatile solvents can evaporate when syringe reactors are heated. Small glass vials with a PTFE septum are a suitable alternative. Transparent adhesive tape is used to secure them and keep them upright in the shaker. Copyright © 2025, photos were recorded by Marius Werner and Truc Lam Pham.

4 Experimental Setup for On-Resin Photocatalysis

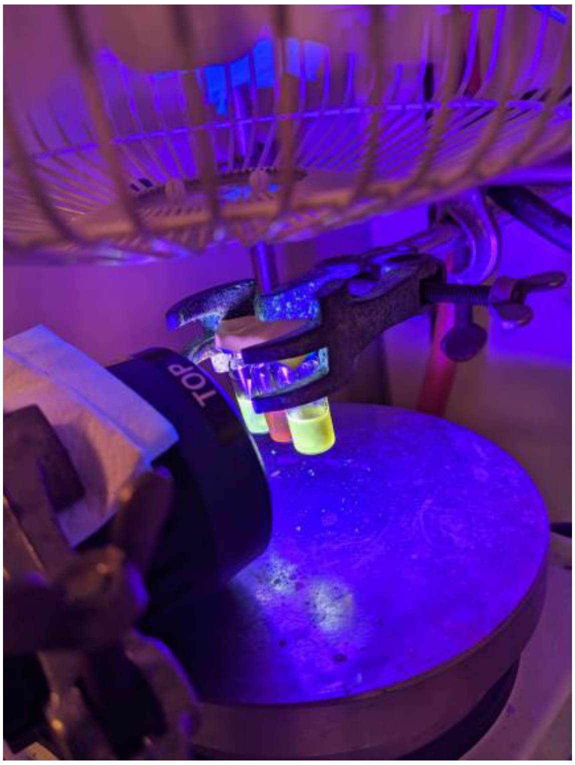

Figure S5: Photoreactor for on resin late-stage functionalization. The reaction mixtures are placed in small glass vials with PTFE stirring bars on a stirring plate and irradiated with a suitable light source. Since irradiation can cause the samples to heat up, a fan is used to maintain room temperature. Adapted from Pal et al.[13] Copyright © 2023 The Authors, under a CC-BY 4.0 license: https://creativecommons.org/licenses/by/4.0/. Published by American Chemical Society.

5 Screening using well plates

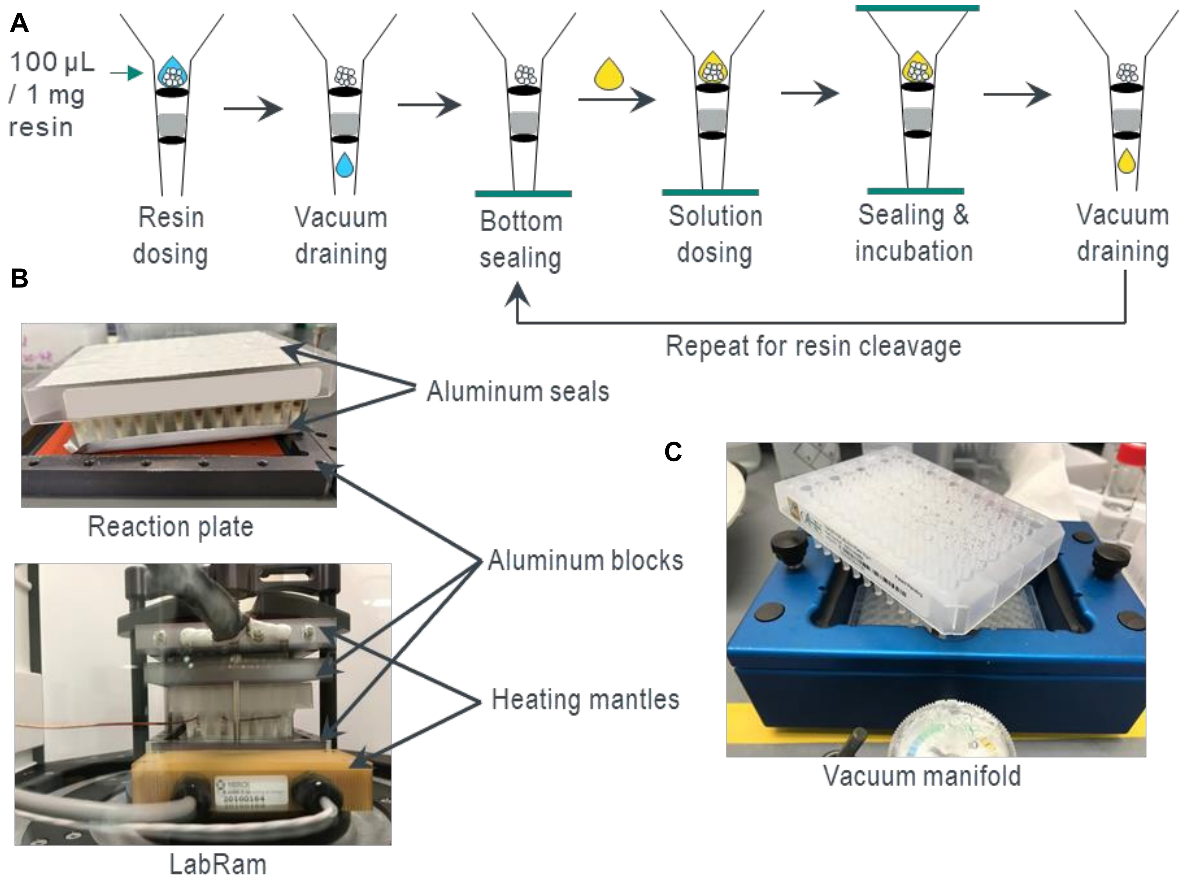

Figure S6: High-throughput late-stage functionalization on the solid phase using 96-well solid-phase extraction plates. A) Each well contains a frit to retain the resin, which can be added using micropipettes as a suspension in a solvent of choice. After draining and sealing the bottom of the plate, reagents can be added and the top of the plate sealed. B) The sealed 96-well plate is placed in a shaker with a heating function. C) After incubation, the seal is removed and the reagents are extracted under vacuum. Adapted with permission from Li et al.[23], Copyright © 2022 American Chemical Society.

6 Literature

[1] D. s. M. M. Jaradat, O. Al Musaimi, F. Albericio, *Advances in solid-phase peptide synthesis in aqueous media (ASPPS)*, *Green Chemistry* **2022**, *24*, 6360-6372.

[2] A. Isidro-Llobet, M. Álvarez, F. Albericio, *Amino Acid-Protecting Groups*, *Chemical Reviews* **2009**, *109*, 2455-2504.

[3] Stephen B. H. Kent, *Fundamental Aspects of SPPS and Green Chemical Peptide Synthesis*, *Journal of Peptide Science* **2025**, *31*, e70013.

[4] O. Al Musaimi, B. G. de la Torre, F. Albericio, *Greening Fmoc/tBu solid-phase peptide synthesis*, *Green Chemistry* **2020**, *22*, 996-1018.

[5] V. Martin, P. H. G. Egelund, H. Johansson, S. Thordal Le Quement, F. Wojcik, D. Sejer Pedersen, *Greening the synthesis of peptide therapeutics: an industrial perspective*, *RSC Advances* **2020**, *10*, 42457-42492.

[6] K. G. Varnava, V. Sarojini, *Making Solid-Phase Peptide Synthesis Greener: A Review of the Literature*, *Chemistry – An Asian Journal* **2019**, *14*, 1088-1097.

[7] A. El-Faham, F. Albericio, *Peptide Coupling Reagents, More than a Letter Soup*, *Chemical Reviews* **2011**, *111*, 6557-6602.

[8] D. Hudson, *Matrix Assisted Synthetic Transformations: A Mosaic of Diverse Contributions. I. The Pattern Emerges*, *Journal of Combinatorial Chemistry* **1999**, *1*, 333-360.

[9] J. Lu, P. H. Toy, *Organic Polymer Supports for Synthesis and for Reagent and Catalyst Immobilization*, *Chemical Reviews* **2009**, *109*, 815-838.

[10] C. Blackburn, *Polymer supports for solid-phase organic synthesis*, *Peptide Science* **1998**, *47*, 311-351.

[11] F. Garcia, F. Albericio, *Solid supports for the synthesis of peptides*, *Chimica Oggi* **2008**, *26*, 29.

[12] J. Brinkhofer, M. Werner, A. Kokollari, S.-Y. Pan, C. Klein, T. L. Pham, F. Thomas, *Late-Stage Amination of Peptides on the Solid Phase*, *Chemistry – A European Journal* **2025**, *31*, e202501229.

[13] S. Pal, J. Openy, A. Krzyzanowski, A. Noisier, P. ‘t Hart, *On-Resin Photochemical Decarboxylative Arylation of Peptides*, *Organic Letters* **2024**, *26*, 2795-2799.

[14] M. Werner, J. Brinkhofer, L. Hammermüller, T. Heim, T. L. Pham, J. Huber, C. Klein, F. Thomas, *Peptide Boronic Acids by Late-Stage Hydroboration on the Solid Phase*, *Advanced Science* **2024**, *11*, 2400640.

[15] N. Jung, M. Wiehn, S. Bräse, *Multifunctional Linkers for Combinatorial Solid Phase Synthesis*, in *Combinatorial Chemistry on Solid Supports* (Ed.: S. Bräse), Springer Berlin Heidelberg, Berlin, Heidelberg, **2007**, pp. 1-88.

[16] J. Alsina, F. Albericio, *Solid-phase synthesis of C-terminal modified peptides*, *Peptide Science* **2003**, *71*, 454-477.

[17] M. Soural, J. Hlaváč, V. Krchňák, *Linkers for Solid-Phase Peptide Synthesis*, in *Amino Acids, Peptides and Proteins in Organic Chemistry*, **2010**, pp. 273-312.

[18] S. Noki, B. G. de la Torre, F. Albericio, *Safety-Catch Linkers for Solid-Phase Peptide Synthesis*, *Molecules* **2024**, *29*, 1429.

[19] M. Economidou, N. Mistry, K. M. P. Wheelhouse, D. M. Lindsay, *Palladium Extraction Following Metal-Catalyzed Reactions: Recent Advances and Applications in the Pharmaceutical Industry*, *Organic Process Research & Development* **2023**, *27*, 1585-1615.

[20] W. P. Gallagher, A. Vo, *Dithiocarbamates: Reagents for the Removal of Transition Metals from Organic Reaction Media*, *Organic Process Research & Development* **2015**, *19*, 1369-1373.

[21] A. Kokollari, M. Werner, C. Lindner, T. L. Pham, F. Thomas, *Rapid On-Resin N-Formylation of Peptides as One-Pot Reaction*, *ChemBioChem* **2023**, *24*, e202300571.

[22] T. Qin, L. R. Malins, J. T. Edwards, R. R. Merchant, A. J. E. Novak, J. Z. Zhong, R. B. Mills, M. Yan, C. Yuan, M. D. Eastgate, P. S. Baran, *Nickel-Catalyzed Barton Decarboxylation and Giese Reactions: A Practical Take on Classic Transforms*, *Angewandte Chemie International Edition* **2017**, *56*, 260-265.

[23] S. Li, D. Pissarnitski, T. Nowak, M. Wleklinski, S. W. Krska, *Merging Late-Stage Diversification with Solid-Phase Peptide Synthesis Enabled by High-Throughput On-Resin Reaction Screening*, *ACS Catalysis* **2022**, *12*, 3201-3210.

[24] D. Orain, J. Ellard, M. Bradley, *Protecting Groups in Solid-Phase Organic Synthesis*, *Journal of Combinatorial Chemistry* **2002**, *4*, 1-16.

[25] S. V. Moradi, W. M. Hussein, P. Varamini, P. Simerska, I. Toth, *Glycosylation, an effective synthetic strategy to improve the bioavailability of therapeutic peptides*, *Chemical Science* **2016**, *7*, 2492-2500.

[26] M. Muttenthaler, F. Albericio, P. E. Dawson, *Methods, setup and safe handling for anhydrous hydrogen fluoride cleavage in Boc solid-phase peptide synthesis*, *Nature Protocols* **2015**, *10*, 1067-1083.
[27] R. Behrendt, P. White, J. Offer, *Advances in Fmoc solid-phase peptide synthesis*, *Journal of Peptide Science* **2016**, *22*, 4-27.
[28] G. B. Fields, R. L. Noble, *Solid phase peptide synthesis utilizing 9-fluorenylmethoxycarbonyl amino acids*, *International Journal of Peptide and Protein Research* **1990**, *35*, 161-214.
[29] S. Knauer, N. Koch, C. Uth, R. Meusinger, O. Avrutina, H. Kolmar, *Sustainable Peptide Synthesis Enabled by a Transient Protecting Group*, *Angewandte Chemie International Edition* **2020**, *59*, 12984-12990.
[30] X. Sun, F. Ye, D. Hu, P. Wang, *Sustainable Peptide Synthesis by Photoredox-Catalyzed Picoc-SPPS*, *Journal of the American Chemical Society* **2025**, *147*, 48244-48253.
[31] M. Amblard, J.-A. Fehrentz, J. Martinez, G. Subra, *Methods and protocols of modern solid phase peptide synthesis*, *Molecular Biotechnology* **2006**, *33*, 239-254.
[32] K. P. Nandhini, M. Alhassan, C. G. L. Veale, F. Albericio, B. G. de la Torre, *Methionine-Containing Peptides: Avoiding Secondary Reactions in the Final Global Deprotection*, *ACS Omega* **2023**, *8*, 15631-15637.
[33] S. N. Mthembu, A. Chakraborty, R. Schönleber, F. Albericio, B. G. de la Torre, *TFA Cleavage Strategy for Mitigation of S-tButylated Cys-Peptide Formation in Solid-Phase Peptide Synthesis*, *Organic Process Research & Development* **2025**, *29*, 691-703.
[34] R. J. Spears, C. McMahon, V. Chudasama, *Cysteine protecting groups: applications in peptide and protein science*, *Chemical Society Reviews* **2021**, *50*, 11098-11155.
[35] A. Chakraborty, S. N. Mthembu, B. G. de la Torre, F. Albericio, *Ready to Use Cysteine Thiol Protecting Groups in SPPS, A Practical Overview*, *Organic Process Research & Development* **2024**, *28*, 26-45.
[36] M. Werner, J. Pampel, T. L. Pham, F. Thomas, *Late-Stage Functionalisation of Peptides on the Solid Phase by an Iodination-Substitution Approach*, *Chemistry – A European Journal* **2022**, *28*, e202201339.
[37] P. Grieco, P. M. Gitu, V. J. Hruby, *Preparation of 'side-chain-to-side-chain' cyclic peptides by Allyl and Alloc strategy: potential for library synthesis*, *The Journal of Peptide Research* **2001**, *57*, 250-256.
[38] K. R. Wilson, S. Sedberry, R. Pescatore, D. Vinton, B. Love, S. Ballard, B. C. Wham, S. K. Hutchison, E. J. Williamson, *Microwave-assisted cleavage of Alloc and Allyl Ester protecting groups in solid phase peptide synthesis*, *Journal of Peptide Science* **2016**, *22*, 622-627.
[39] P. Napier, N. Bakas, A. Bhat, A. Noncovich, *Open-Flask Protocol for the Removal of Alloc Carbamate and Allyl Ester Protecting Groups. Application to In-solution and On-resin Peptide Synthesis*, *The Journal of Organic Chemistry* **2025**, *90*, 197-201.
[40] J. Pawlas, J. Billing, B. Tebikachew, L. Wahlström, L. M. Haugaard-Kedström, *A Sustainable Approach to ε-Lys Branched GLP-1 Analogs: Integrating Green SPPS, Metal-free Alloc Removal, Waste Minimization and TFA/PFAS-free Resin Cleavage*, *Organic Process Research & Development* **2025**, *29*, 2989-2997.
[41] J. Pawlas, A. Lindgren, *Expanding the Reach of Sustainable Solid-Phase Peptide Synthesis: One-Pot, Metal-Free Alloc Removal–Peptide Coupling*, *Organic Letters* **2025**, *27*, 2891-2896.
[42] S. R. Chhabra, B. Hothi, D. J. Evans, P. D. White, B. W. Bycroft, W. C. Chan, *An appraisal of new variants of Dde amine protecting group for solid phase peptide synthesis*, *Tetrahedron Letters* **1998**, *39*, 1603-1606.
[43] T. Bruckdorfer, O. Marder, F. Albericio, *From Production of Peptides in Milligram Amounts for Research to Multi-Tons Quantities for Drugs of the Future*, *Current Pharmaceutical Biotechnology* **2004**, *5*, 29-43.
[44] W. Tang, M. L. Becker, *"Click" reactions: a versatile toolbox for the synthesis of peptide-conjugates*, *Chemical Society Reviews* **2014**, *43*, 7013-7039.
